# Structural basis for mitoguardin-2 mediated lipid transport at ER-mitochondrial membrane contact sites

**DOI:** 10.1038/s41467-022-31462-6

**Published:** 2022-06-28

**Authors:** Hyunwoo Kim, Seowhang Lee, Youngsoo Jun, Changwook Lee

**Affiliations:** 1 Department of Biological Sciences, Ulsan National Institute of Science and Technology, Ulsan, Korea; 2 Cell Logistics Research Center, Gwangju Institute of Science and Technology, Gwangju, Korea; 3 School of Life Sciences, Gwangju Institute of Science and Technology, Gwangju, Korea

**Keywords:** Transport carrier, Phospholipids, Mitochondria, Endoplasmic reticulum, X-ray crystallography

## Abstract

The endoplasmic reticulum (ER)-mitochondria contact site (ERMCS) is crucial for exchanging biological molecules such as phospholipids and Ca^2+^ ions between these organelles. Mitoguardin-2 (MIGA2), a mitochondrial outer membrane protein, forms the ERMCS in higher eukaryotic cells. Here, we report the crystal structures of the MIGA2 Lipid Droplet (LD) targeting domain and the ER membrane protein VAPB bound to the phosphorylated FFAT motif of MIGA2. These structures reveal that the MIGA2 LD targeting domain has a large internal hydrophobic pocket that accommodates phospholipids and that two phosphorylations of the FFAT motif are required for tight interaction of MIGA2 with VAPB, which enhances the rate of lipid transport. Further biochemical studies show that MIGA2 transports phospholipids between membranes with a strong preference for binding and trafficking phosphatidylserine (PS). These results provide a structural and molecular basis for understanding how MIGA2 mediates the formation of ERMCS and facilitates lipid trafficking at the ERMCS.

## Abstract (web summary)

The ER-mitochondria contact sites are crucial for exchanging phospholipids. Here, Kim et al. present crystal structures of mitoguardin-2 (MIGA2) which reveal that MIGA2 directly binds phospholipids and transfers them between the ER and mitochondria.

## Introduction

In eukaryotic cells, subcellular compartments contact each other through membrane contact sites (MCSs)^1–3^. The MCS is formed mainly by intermolecular interactions between proteins from each organelle, and MCS-tethering complexes play a pivotal role in non-vesicular trafficking of lipids, ions, and metabolites. The ER-mitochondria MCS (ERMCS) is well established among other MCSs, and a range of tethering proteins have been reported^1,3–9^. The ERMCS functions in controlling mitochondria metabolism as well as Ca^2+^ signaling^5^. In particular, it plays an important role in the maintenance of lipid homeostasis and distribution by facilitating local lipid synthesis and non-vesicular transfer between the ER and mitochondria membranes^10^. In yeast, the ERMCS is formed by a hetero-tetrameric ER-mitochondrial-encounter structure (ERMES) complex comprising mitochondrial distribution and morphology protein 12 (Mdm12), maintenance of mitochondrial morphology 1 (Mmm1), Mdm34, and Mdm10^11^. Among these, Mdm12, Mmm1, and Mdm34 each contain a characteristic synaptotagmin-like mitochondrial-lipid-binding (SMP) domain that facilitates binding and trafficking of phospholipids between the ER and the mitochondrion^12–15^. In metazoans, while no functional hetero-complexes like the ERMES have been found, the PDZ domain-containing protein 8 (PDZD8) was first identified as a mammalian ortholog of yeast Mmm1^16^. In fact, primary structure analysis has revealed that PDZD8 contains the SMP domain, suggesting the possibility of lipid trafficking at the ERMCS. However, a recent study suggested that PDZD8 might be a paralog rather than a functional ortholog of Mmm1, and that it is involved in lipid trafficking at the ER-plasma membrane (PM), ER-late endosome, and ER-lysosome contact sites^17,18^.

In addition, the mitochondria outer membrane protein, protein tyrosine phosphatase interacting protein 51 (PTPIP51), was found to be a molecular tether linking the ER and mitochondria in higher eukaryotes^19^. The PTPIP51 is recruited to the ERMCS by binding vesicle-associated membrane protein-associated protein B/C (VAPB), an ER membrane protein. The resultant VAPB-PTPIP51 complex functions in the regulation of Ca^2+^ homeostasis, autophagy, as well as synaptic activity^20,21^. The tetratricopeptide repeat (TPR) domain of PTPIP51 transfers a phosphatidic acid (PA) in vitro^22^. Recent studies have found that oxysterol-binding protein-related proteins 5 and 8 (ORP5 and ORP8), known at ER-PM contacts sites as PS and phosphatidylinositol 4-phosphate (PI4P) exchangers, could be recruited to mitochondria by interacting with PTPIP51 and might mediate PS trafficking^23,24^. However, it is not yet known how phospholipids other than PA and PS are transferred by non-vesicular trafficking between the ER and mitochondria in higher eukaryotic cells. Other mammalian ERMCS tethering proteins such as the voltage-dependent anion channel 1 (VDAC1)-inositol trisphosphate receptor (IP3R), mitochondrial fission 1 homolog (FIS1)-B-cell receptor-associated protein 31 (BAP31), and mitofusin 2 (MFN2) have been reported, but none of these are known to be directly involved in lipid trafficking^25–27^.

A recent study reported that MIGA2, an outer mitochondrial membrane protein, promotes synthesis of triglycerides by linking mitochondria to the ER and lipid droplets (LDs) in diverse cell types including adipocytes^28^. MIGA2 interacts directly with VAP-A or VAP-B to mediate the ERMCS^28^. Another study revealed that overexpression of Miga increases the formation of ERMCS and causes severe neurodegeneration in fly^29^. Mattia and colleagues recently revealed that MIGA2 has a non-conventional FFAT (two phenylalanines in an acidic tract) motif in which a conserved acidic residue is replaced by a serine/threonine^30^. The interaction with VAPB requires phosphorylation of the FFAT motif of MIGA2^29,30^. Comparative sequence analysis revealed that the MIGA is conserved from nematode to human and no equivalent homologs are found in yeast^29^. Mammalian cells have two MIGA paralogs (MIGA1 and MIGA2) that share 36.1% sequence identity. Interestingly, knock out mice of MIGA1/2 reduces body fat and hepatic lipidosis, indicating that MIGA might function in lipid metabolism^31,32^. Based on these findings, it has been proposed that MIGA2 might be an important player in the trafficking of lipids at the ERMCS^28,29,31^. However, the molecular mechanism via which MIGA2 contributes to lipid trafficking at the ERMCS remains to be investigated. In addition, it remains largely unknown how phosphorylation of the non-conventional FFAT motif in MIGA2 affects recognition by VAPB, and thus it is unclear how it may contribute to membrane tethering.

Here, we report the crystal structures of the MIGA2 LD targeting domain and the major sperm protein (MSP) domain of VAPB bound to the phosphorylated FFAT motif of MIGA2. Unexpectedly, the structure of the MIGA2 LD targeting domain reveals an unidentified glycerophospholipid with two long hydrocarbon chains inside a large cavity. Biochemical experiments using liposomes demonstrate that MIGA2 specifically transfers a PS between two membranes. Furthermore, the structure of the phosphorylated FFAT-VAPB complex reveals that MIGA2 mediates formation of the ERMCS and thereby facilitates lipid trafficking at the ERMCS in higher eukaryotic cells.

## Results

### MIGA2 directly associates with liposomes and glycerophospholipids

**Fig. 1:**
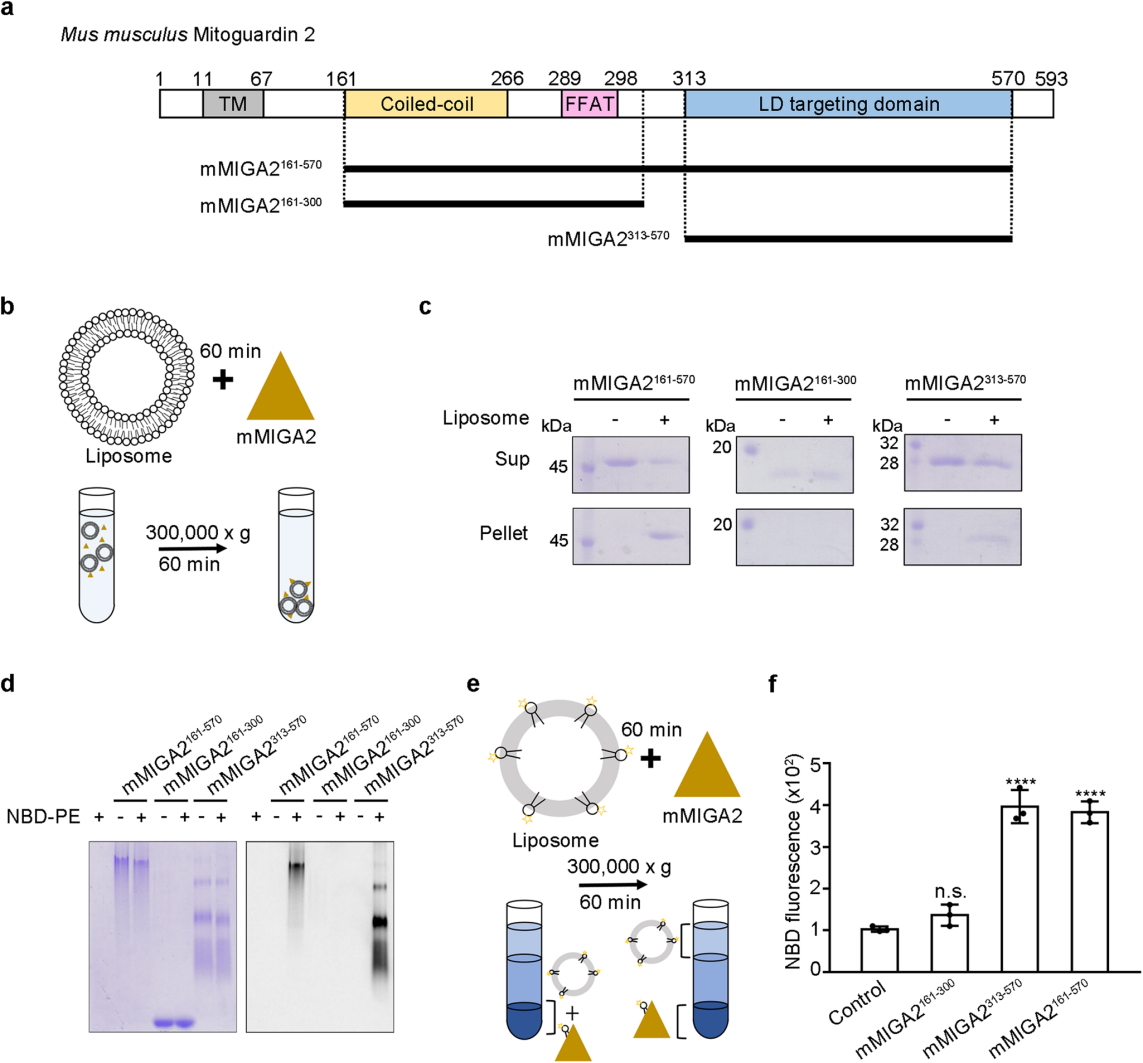
MIGA2 directly binds to phospholipids. **a** Diagram showing the domain structure of *Mus musculus* MIGA2. From the N-terminus, the MIGA2 has a transmembrane domain (TM), Coiled-coil domain, FFAT motif, and LD targeting domain. **b** Schematic diagram of the liposome sedimentation assay. The mMIGA2 fragments were incubated with liposomes for 60 min and separated by centrifugation. **c** Figure showing the results of the liposome-binding assay performed as described in **b**. Supernatant (Sup) and precipitant fractions (Pellet) were analyzed by 12% SDS-PAGE and Coomassie blue staining (*N* = 2 independent experiments). **d** The fluorescence lipid-binding assay. The purified mMIGA2 proteins were incubated with NBD-PE and subjected to 10% Clear Native-PAGE (CN-PAGE) (*N* = 2 independent experiments). Coomassie staining (left) and fluorescent detection (right). **e** Diagram showing the in vitro lipid extraction assay. Liposomes containing NBD-PE were incubated with the mMIGA2 for 60 min. The mixtures were separated by centrifugation and precipitated fractions were analyzed by a fluorescence measurement. **f** Bar graph showing the results of lipid extraction by mMIGA2 using experiments performed as described in **e**. Lipid extraction activity was compared to that of the control using one-way ANOVA (*N* = 3 independent experiments; individual data point shown as dots, bars show mean ± SD). ****p < 0.0001 (mMIGA2^161–570^ and mMIGA2^313–570^). Source data are provided in a Source data file.

Mammalian MIGA2 has several distinct domains: a mitochondrial targeting transmembrane domain (TM) at the N-terminus, a coiled-coil domain, the FFAT motif, and a large C-terminal LD targeting domain (Fig. 1a). MIGA2 is anchored in the mitochondrial outer membrane via the TM, and associates with the ER via a direct interaction between its FFAT motif and VAPA/B, an ER membrane protein^28^. MIGA2 forms contact sites between these organelles and LDs through a predicted LD targeting domain comprising residues 450–550^28^. Given this background information, we first explored whether MIGA2 binds membranes in vitro. For this, we generated three truncated recombinant constructs of mouse MIGA2 (mMIGA2) as indicated in Fig. 1a, and prepared purified protein (Supplementary Fig. 1). The proteins were then incubated with liposomes for 60 min, and the precipitated liposomes were analyzed by SDS-PAGE (Fig. 1b). Figure 1c shows that the MIGA2 LD targeting domain (mMIGA2^313–570^) associated with liposomes, whereas the central MIGA2 domain (mMIGA2^161–300^), consisting of the coiled-coil domain and FFAT motif, did not. Next, we tested whether MIGA2 binds individual glycerophospholipids using NBD-PE, a fluorescence-labeled phosphatidylethanolamine (PE). Protein-NBD-PE mixtures were separated by Clear Native-PAGE (CN-PAGE) and detected by fluorescence. Consistent with the liposome binding experiments, mMIGA2^313–570^, but not mMIGA2^161–300^, bound to NBD-PE (Fig. 1d). To clarify whether MIGA2 is involved in extracting phospholipids from liposomes, we performed lipid extraction as described in a previous study^12,15^. The results confirmed that mMIGA2^313–570^ can extract NBD-PE from liposomes (Fig. 1e, f). Collectively, these biochemical results suggest that the LD targeting domain of MIGA2 enables MIGA2 proteins to traffic lipids at their membrane contact regions.

### Overall structure of zMiga2

To understand the molecular mechanism by which MIGA2 facilitates trafficking of glycerophospholipids at membranes, we attempted to solve the structure of murine MIGA2. Unfortunately, crystallization of full-length mouse MIGA2 was not successful; thus, we crystallized zebra fish Miga2 instead (zebra fish Miga2 shares 61.2% and 60.83% sequence identity to human and mouse MIGA2, respectively). We obtained crystals of the zebra fish Miga2 LD targeting domain (residues 310–568), which we refer to as zMiga2. zMiga2 shares 74.9% and 76.1% sequence identity to human and mouse LD targeting domains, respectively. A crystal of zMiga2 diffracted X-rays to 2.85 Å resolution, and the structure was solved by single-wavelength anomalous dispersion (SAD) using selenium as an anomalous scatterer (Supplementary Fig. 2 and Supplementary Table 1).

**Fig. 2:**
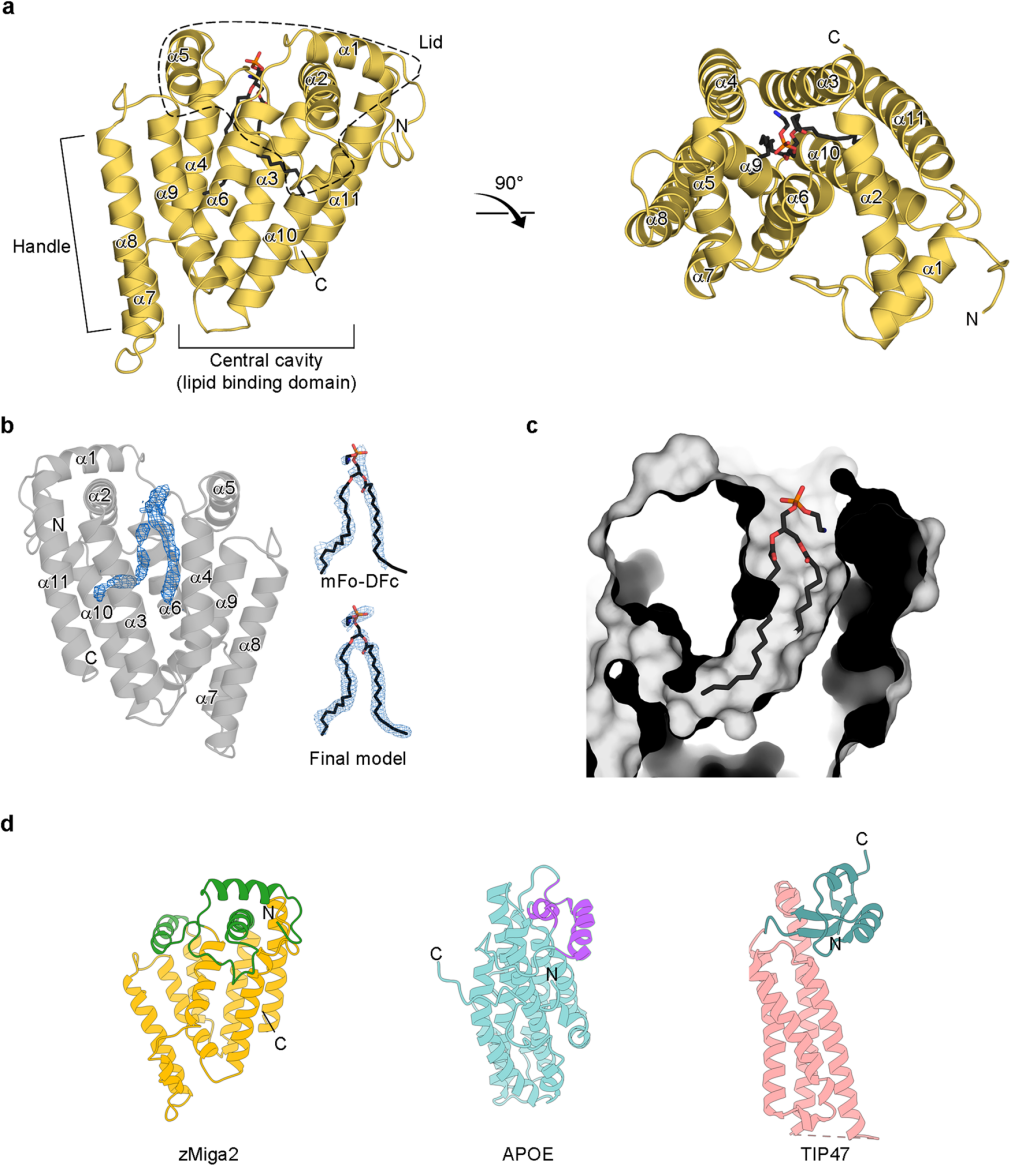
Overall structure of zMiga2. **a** Ribbon representation of the zMiga2 (yellow orange). The structure was determined by Se-SAD phasing and refined to 2.85 Å resolution. The overall structure is reminiscent of a mug. The lid is highlighted by dashed lines. The handle protrudes on the left. The black stick model indicates the phospholipid bound to zMiga2. Phosphate, nitrogen, and oxygen atoms are colored in orange, blue, and red, respectively. **b** The crystal structure of zMiga2 revealed extra electron density (blue, 2.85 Å resolution, contoured at 1.5 σ), which seemed to be that of a glycerophospholipid (left). Based on the density, we built a PE model with two 16:0 hydrocarbon chains. The final model is shown with the fo-fc map (right top) and 2fo-fc map (right below). The fo-fc map was calculated in the absence of the lipid model and countered at 1.5 σ. The 2fo-fc map is contoured at 0.8 σ. **c** A protein surface cutaway view shows a large cavity in zMiga2. The structure of PE is shown as a stick representation. **d** A ribbon diagram shows a structural comparison of lid and helix bundle structure of zMiga2 (lid: green; helix bundle: yellow orange), APOE (lid: purple; helix bundle: cyan) (PDB ID: 2L7B), and TIP47 (lid: teal; helix bundle: pink) (PDB ID: 1SZI).

The zMiga2 structure resembles a “mug” and consists of eleven α-helices (Fig. 2a and Supplementary Fig. 3). The structure comprises three major features: a mug surface-like structure made up of central helices (α3, α4, α6, α9, α10, and α11) with a large inside cavity; a mug lid-like structure made up of α1, α2, α5 helices and a long loop between α1 and α2 arranged in a perpendicular manner relative to the axes of the central face helices, and a mug handle-like structure made up of a pair of α8 and relatively short α7 helices protruding in parallel from the central cavity. The central six helices of the mug surround a large cavity of approximately 1100 Å^3^ (Fig. 2c and Supplementary Fig. 4a)^33^. Intriguingly, inside the cavity, we found extra electron density that seemed to correspond to that of a glycerophospholipid, although no glycerophospholipids were added during protein preparation or crystallization. We modeled this as PE with two 16:0 fatty acyl chains, since PE is the most abundant species in bacteria cells cultured in LB media^34^. The lipid structure refined well with temperature factors of around 40–45 Å^2^ (Fig. 2b, c). Due to disordered electron density around the head group of the phospholipid, we could not identify it accurately.

Comparative analyses against structures in the Protein Data Bank (PDB) using the Dali server^35^ revealed that the structures of LDL receptor-binding domain of apolipoprotein E (APOE) and C-terminal domain of tail-interacting protein of 47 kD (TIP47) resembled that of zMiga2^35^. Both structures share the overall structural features of a helix bundle at the center with a lid of perpendicular helices (Fig. 2d and Supplementary Fig. 5). APOE binds lipid and functions in lipid transport between subcellular organs in eukaryotic cells^36,37^, and TIP47 is found in LDs and the cytosol, and is involved in lipid metabolism^38^. A previous study suggested that the central helix bundle of APOE and TIP47 might interact with lipids^36–38^. This structural conservation supports the notion that the LD targeting domain of MIGA2 also plays a role in trafficking glycerophospholipids.

### zMiga2 has a large hydrophobic cavity for lipid binding

**Fig. 3:**
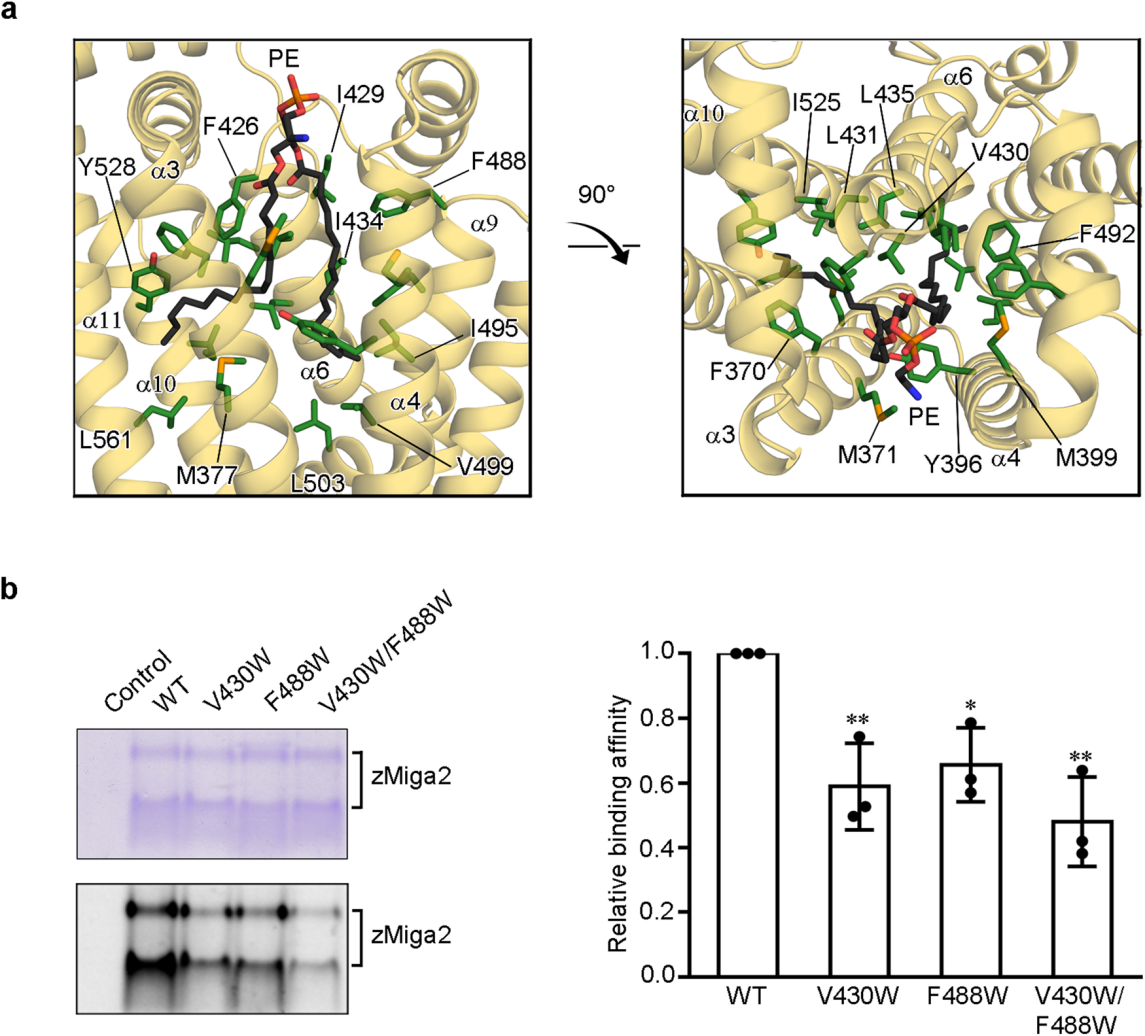
MIGA2 binds glycerophospholipid in a large hydrophobic cavity. **a** Close-up views highlight the interactions between zMiga2 (yellow orange) and phospholipid (black). The zMiga2 residues involved in the interaction with phospholipid are shown using a green stick model. The remaining color scheme is the same as in Fig. 2a. **b** The figures show the lipid-binding assay for zMiga2 WT and its mutants (V430W, F488W, and V430W/F488W). Proteins incubated with NBD-PE were subjected to 14% CN-PAGE, and analyzed by Coomassie staining (left-top) and fluorescence (left-below). The bar graph on the right shows quantification of the data obtained in this assay. The lipid-binding activities of the mutants were compared with that of WT using one-way ANOVA (*N* = 3 independent experiments; individual data point shown as dots, bars show mean ± SD). **p = 0.0051 (V430W), *p = 0.0137 (F488W), **p = 0.0012 (V430W/F488W). Source data are provided in a Source data file.

The structure of zMiga2 revealed a hydrophobic cavity surrounded by the six central helices of the ‘mug’. The helices are tilted around 10–25 degrees away from the mug vertical axis, converging at the bottom of the mug in a closed structure (Fig. 2a). The other end is relatively open, which would facilitate anchoring of the head group of a glycerophospholipid. The central helices are amphiphilic with the hydrophobic sides facing inward; thus, the cavity is hydrophobic and can accommodate the hydrocarbon chains of a glycerophospholipid (Fig. 3a). Side chains of helices α3 (F370, M371, and M377), α6 (F426 and L431), α10 (I525 and Y528), and α11 (L561) make hydrophobic interactions with the first hydrocarbon chain. The hydrophobic side chains of helices α4 (Y396 and M399), α6 (I429 and I434), and α9 (F488, F492, I495, V499, and L503) contact the second hydrocarbon chain. V430 and L435 of α6 interact with both hydrocarbon chains (Fig. 3a). All residues involved in these interactions are highly conserved among other species, indicating that lipid binding by MIGA2 is evolutionary conserved (Supplementary Fig. 3). We did not observe any electrostatic or hydrogen-bonding interactions between the glycerophospholipid head group and zMiga2, suggesting that the lipid interaction is purely hydrophobic in nature.

To confirm whether the above-mentioned residues contribute to interactions with lipids in solution, we introduced tryptophan mutations at these residues to confer steric hindrance and explored the ability of the mutant proteins to interact with NBD-PE^13^. CD spectroscopy revealed no difference between the spectrum of wild-type zMiga2 and the spectra of the mutants, indicating that the mutations did not affect the overall conformation of zMiga2 (Supplementary Fig. 6a). Although the mutations also did not affect liposome binding (Supplementary Fig. 6b), the V430W or F488W single mutant showed a slight decrease in NBD-PE binding, and the V430W and F488W double mutant showed decreased lipid-binding that was less than half of that of the wild type (Fig. 3b). These results suggested that the LD targeting domain of MIGA2 is specifically designed to bind glycerophospholipids through hydrophobic interactions in solution.

### MIGA2 prefers to transfer phosphatidylserine

**Fig. 4:**
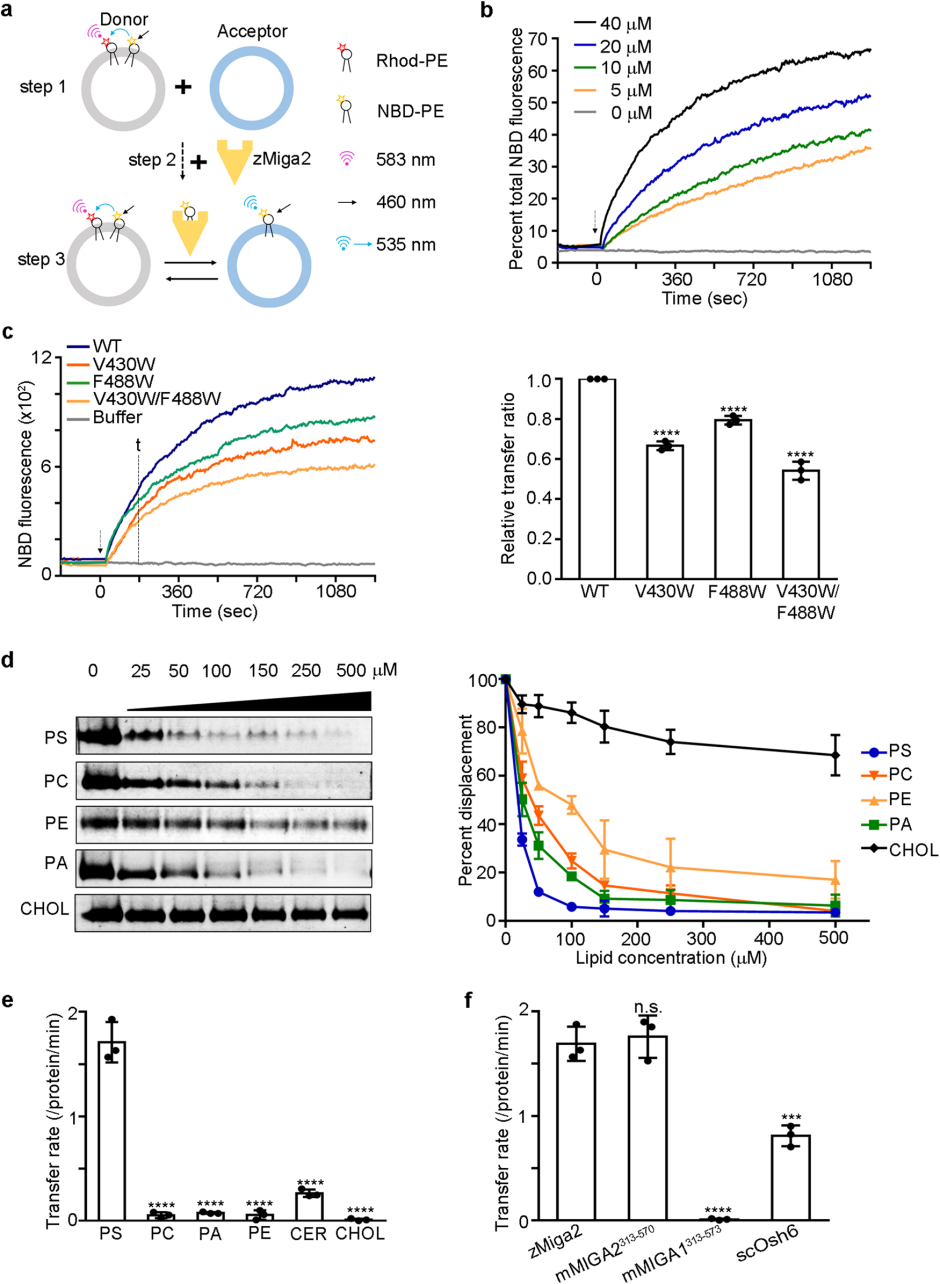
MIGA2 prefers to transfer PS. **a** Schematic diagram showing the lipid transfer assay. Briefly, donor liposomes containing both NBD-PE and Rhod-PE and fluorescence-labeled free acceptor liposomes were prepared. NBD-PE fluorescence was quenched by Rhod-PE in the donor liposomes (step 1). Then, zMiga2 was added to the mixture of donor and acceptor liposomes (step 2), and the lipid transfer activity was measured by observing increases in the fluorescence signal at 535 nm (step 3). **b** In vitro lipid transfer assay. The graph shows time courses for fluorescence emitted by NBD-PE in concentration-dependent reactions. The fluorescence output of NBD-PE transferred from the donor liposome (50 µM) to the acceptor liposome (50 µM) was monitored upon addition of various concentrations of zMiga2. The arrow indicates the point of protein injection. **c** In vitro lipid transfer assay. The NBD fluorescence was measured after addition of wild-type zMiga2 or its mutants (V430W, F488W, and V430W/F488W). The time point “*t*” indicates the time used to calculate the relative lipid transfer ratio. Lipid transfer ratio was compared with that of WT using one-way ANOVA (*N* = 3 independent experiments; individual data point shown as dots, bars show mean ± SD). ****p < 0.0001 (V430W, F488W, and V430W/F488W). **d** In vitro phospholipid displacement experiment using NBD-PC. The NBD-PC preloaded zMiga2-GST was mixed with natural phospholipids (PS, PC, PE, and PA) and cholesterol (CHOL) at increasing concentrations, and the quantify of NBD-PC displaced by natural ligand was analyzed by observing decreases in fluorescence. The right-hand graph indicates quantification data using left (*N* = 3 independent experiments; dot shows mean ± SD). **e** The graph summarizes the transfer rates of lipid transfer by 100 nM of zMiga2 for a series of phospholipids (PS, PC, PE, and PA), ceramide (CER), and CHOL substrates. Lipid transfer activity was compared with that of PS using one-way ANOVA (*N* = 3 independent experiments; individual data point shown as dots, bars show mean ± SD). ****p < 0.0001 (PC, PA, PE, CER, and CHOL). **f** The graph compares the rates of lipid transfer by 100 nM of zMiga2, mMIGA2^313–570^, mMIGA1^313–573^, and scOsh6. Lipid transfer activity was compared with that of zMiga2 using one-way ANOVA (*N* = 3 independent experiments; individual data point shown as dots, bars show mean ± SD). ****p < 0.0001 (mMIGA1^313–573^), ***p = 0.0002 (scOsh6). Source data are provided in a Source data file.

We further explored whether the LD targeting domain of MIGA2 can transfer glycerophospholipids across the membrane, since MIGA2 is known to be associated with membranes of mitochondria, ER, and LDs^28^. For this, we performed a lipid transfer assay as described in a previous study^12,39^. First, we prepared a mixture of two liposomes, a donor and acceptor, using rhodamine-PE (Rhod-PE) and NBD-PE (Fig. 4a, step 1). The donor liposome contained both Rhod-PE and NBD-PE while the acceptor liposome did not. Rhod-PE quenches the fluorescence emitted by NBD-PE; therefore, no fluorescence is detected without perturbation. After purified zMiga2 protein was added to the mixture (step 2), lipid transfer was measured by observing an increase in fluorescence (Fig. 4a, step 3). As shown in Fig. 4b, wild-type zMiga2 transferred the NBD-PE from donor liposome to acceptor liposome in a concentration-dependent manner. However, V430W and F488W mutants showed impaired transfer of NBD-PE (Fig. 4c). Interestingly, the effects caused by the mutations were comparable between the lipid binding and lipid transfer experiments. Taken together, the results suggest that zMiga2 can transfer phospholipids by using the hydrophobic cavity of its LD targeting domain.

Next, we examined whether zMiga2 has a preference for transferring specific glycerophospholipids. First, we performed a lipid displacement assay as described in previous studies^13,14^. Purified zMiga2 with GST fused at the C-terminus was incubated with a fluorescence-labeled phosphatidylcholine (PC), NBD-PC, and the mixture was precipitated with Glutathione Sepharose beads to remove free NBD-PC completely. The NBD-PC bound zMiga2 was incubated with natural lipids across a range of concentrations, and the displacement of NBD-PC by natural ligands was measured by observing a decrease in fluorescence. Figure 4d shows that zMiga2 mostly interacted with any glycerophospholipid possessing hydrocarbon chains, which is consistent with the crystal structure. Cholesterol, which has a bulky and rigid structure, could not displace the NBD-PC. It is noteworthy that zMiga2 showed the strongest preference for PS among the other lipids tested (Fig. 4d).

We further probed whether the phospholipid preference of MIGA2 would affect lipid transfer. To this end, we carried out a lipid transfer assay with fluorescence-labeled phospholipids such as NBD-PS, NBD-PC, NBD-PA, NBD-PE, NBD-Ceramide, and NBD-Cholesterol. We observed that zMiga2 transferred PS more rapidly than other phospholipids (Fig. 4e and Supplementary Fig. 7a), which is consistent with the results of the lipid displacement experiments (Fig. 4d). We tested the phospholipid transfer of the mouse MIGA1 LD targeting domain (residues 313–573), a paralog of MIGA2 (mouse MIGA1 LD targeting domain shares 52.1% sequence identity to the mouse MIGA2 LD targeting domain), but no transfer was observed (Fig. 4f and Supplementary Fig. 7b). The MIGA1 LD targeting domain was not defective in liposome binding (Supplementary Fig. 8a). Intriguingly, we observed that NBD-PE binding by MIGA1 was markedly lower than that by MIGA2 (Supplementary Fig. 8b), and that MIGA1 was unable to extract phospholipids from the liposome (Supplementary Fig. 8c). However, both zMiga2 and mMIGA2^313–570^ showed greater transfer than *Saccharomyces cerevisiae* oxysterol-binding protein homolog 6 (scOsh6), a yeast homolog of ORP5, which is a well-known protein involved in PS and PI4P transport between the ER and PM (Fig. 4f and Supplementary Fig. 7b)^24,40–42^.

### Phosphorylated FFAT motif of MIGA2 binds to VAPB

**Fig. 5:**
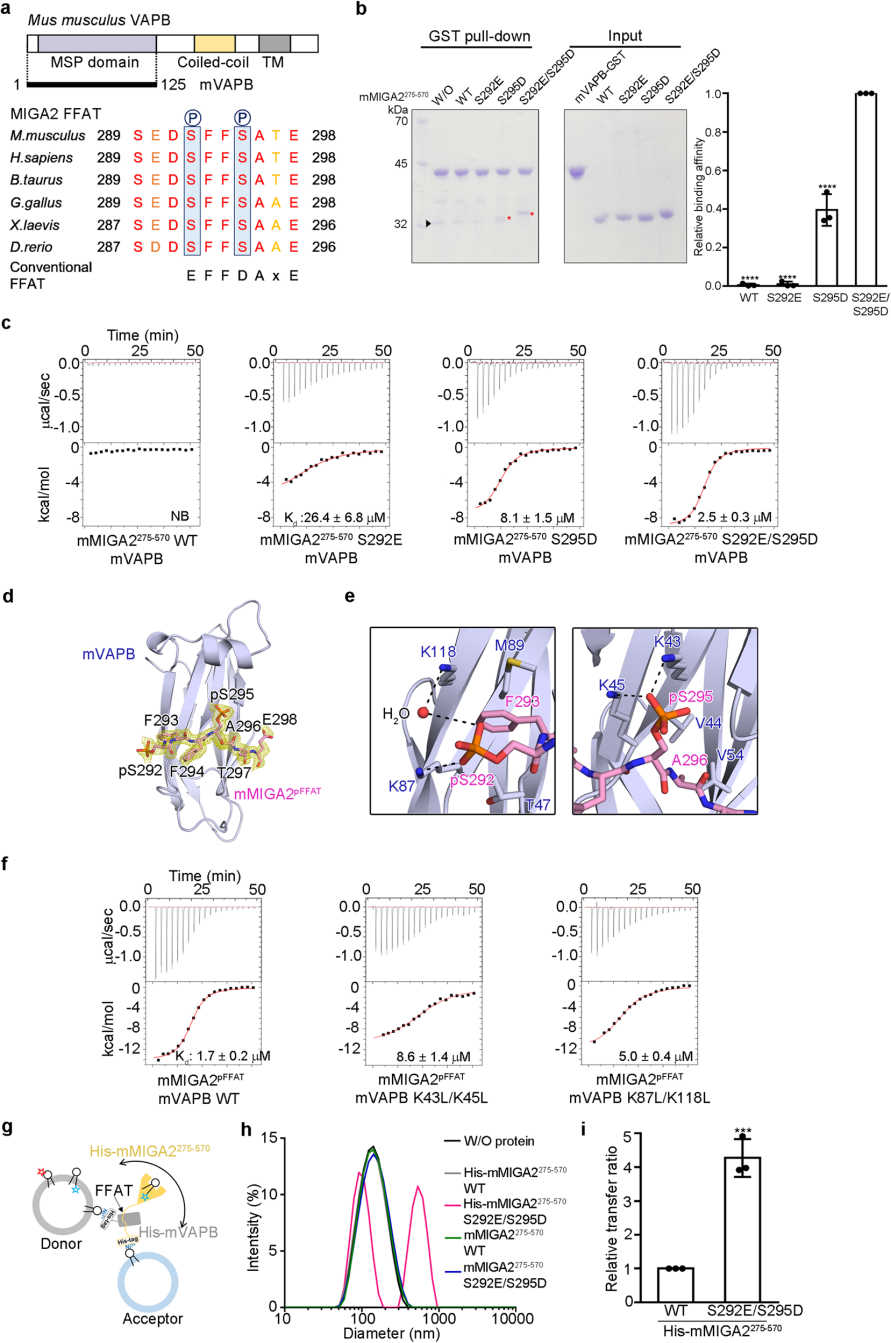
MIGA2 interacts with VAPB via the phosphorylated FFAT motif. **a** Domain structure of mVAPB. The sequence alignment of FFAT motif in MIGA2 orthologs is shown to highlight the sequence conservation. Phosphorylation sites are marked with blue boxes. **b** GST pull-down assay showing the interaction between mMIGA2 and mVAPB, using wild type and phosphorylation mimic mutants of mMIGA2^275–570^. The mVAPB fused to GST was incubated with purified mMIGA2 proteins. Red star and black triangle indicate mMIGA2 bound to mVAPB and the impurity, respectively. The quantification data is shown in the right bar graph. mMIGA2 binding affinity was compared with that of S292E/S295D using one-way ANOVA (*N* = 3 independent experiments; individual data point shown as dots, bars show mean ± SD). ****p < 0.0001 (WT, S292E, and S295D). **c** ITC measurements of mMIGA2^275–570^ wild type and phosphorylation mimic mutant binding to mVAPB. The upper panel shows the primary data. The lower panel shows data fitted to binding isotherms to obtain affinities. **d** Structure of mMIGA2^pFFAT^ bound to mVAPB (blue). The mMIGA2^pFFAT^ is drawn using a stick representation in pink, with oxygen and nitrogen atoms colored red and blue, respectively. The difference density map (fo-fc, yellow) was calculated in the absence of mMIGA2^pFFAT^ (1.68 Å resolution, contoured at 1.6 σ). **e** Close-up view shows that MIGA2 phosphorylated residues are recognized by positively charged residues in mVAPB. The dashed lines and the red sphere indicate intermolecular hydrogen bonds and a bound water molecule, respectively. **f** ITC analysis of the binding affinities of wild type and mutants of the mVAPB to mMIGA2^pFFAT^. **g** Schematic diagram of the lipid transfer assay with membrane-bound His-mMIGA2^275–570^ and His-mVAPB (see details in Methods). **h** DLS analysis showing the formation of a liposome cluster mediated by interaction between the phosphomimetic FFAT motif of mMIGA2^275–570^ and mVAPB. **i** The bar graph shows the effect of phosphorylation of the FFAT motif of mMIGA2^275–570^ (100 nM) on the lipid transfer rate when both the His-mMIGA2^275–570^ and His-mVAPB are associated with the membranes. Lipid transfer activity was compared with that of His-mMIGA2^275–570^ WT using the two-sided *t*-test (*N* = 3 independent experiments; individual data point shown as dots, bar show mean ± SD, ***p = 0.0005). Source data are provided in a Source data file.

It has been shown that MIGA2 can associate with ER membranes via its FFAT motif^28–30^. It is well-known that the FFAT motif is specifically recognized by the MSP domain of VAPA/B^43^. As mentioned earlier, MIGA2 also has the FFAT motif (residues 289–298 in mMIGA2) (Fig. 5a and Supplementary Fig. 9a)^28–30,43–45^. A recent study revealed that phosphorylation of serine residues inside the FFAT motif is required for MIGA2 interaction with VAPB^29,30^. Based on this information, we examined whether phosphorylation of the two conserved serine residues (S292 and S295 in mouse) in the MIGA2 FFAT motif is critical for its interaction with VAPB in vitro (Fig. 5a). We prepared the MSP domain of mouse VAPB (residues 1–125, referred to as mVAPB) tagged with GST and a truncated mMIGA2 fragment comprising residues 275–570 including the FFAT motif (Supplementary Fig. 9b). We also generated phosphorylation mimic mutants (S292E and S295D) of mMIGA2^275–570^, and performed GST pull-down experiments. Wild type mMIGA2^275–570^ was not pulled down by mVAPB (Fig. 5b), whereas the S292E/S295D double mutant was, suggesting that phosphorylation is important for VAPB binding in vitro. The S292E or S295D single-point mutant did not interact with mVAPB as strongly as the double mutant (Fig. 5b). This differs from the VAPA-star-related lipid transfer domain 3 (STARD3) interaction, in which a single phosphorylation is sufficient for VAPA interaction^30^. The importance of phosphorylation of the two conserved serine residues was also confirmed by isothermal titration calorimetry (ITC). ITC measurements demonstrated that wild-type mMIGA2^275–570^ could not interact with mVAPB (Fig. 5c and Supplementary Table 2), whereas the S292E and S295D phosphorylation mimic mutants of mMIGA2^275–570^ showed binding affinities (*K*_d_) of 26.4 ± 6.8 and 8.1 ± 1.5 μM, respectively. The S292E and S295D double mutations synergistically increased the binding affinity to 2.5 ± 0.3 μM (Fig. 5c and Supplementary Table 2), suggesting that both phosphorylations are needed for association with VAPB.

How does the phosphorylation of FFAT motif in MIGA2 contribute to its recognition by VAPB? To answer this question, we solved the crystal structure of mouse VAPB MSP domain bound a phosphorylated FFAT peptide from mMIGA2 (residues 289–298, referred to as mMIGA2^pFFAT^) at 1.68 Å resolution (Fig. 5d and Supplementary Table 1). The overall structure of the mVAPB-mMIGA2^pFFAT^ complex highly resembles that of VAPA-ORP1 (rmsd of 0.74 Å over 108 Cα) and VAPA-STARD3 (rmsd of 0.81 Å over 112 Cα), which possess a conventional and a single phosphorylated FFAT motif, respectively^30,43^. Overall, the extended structure of mMIGA2^pFFAT^ peptide binds across the center of mVAPB. Specifically, the phosphorylated S295 residue makes interactions with the side chains of K43 and K45 residues of mVAPB (Fig. 5e), and these interactions are also observed in the structure of the VAPA-STARD3 complex^30^. The phosphorylated S292 residue forms an ionic bond with the side chain of a conserved K87 residue of mVAPB (Fig. 5e) and also associates with the K118 residue of mVAPB via a water molecule. To test whether these residues are genuinely responsible for mVAPB interactions with mMIGA2^pFFAT^, we generated mutants (K43L/K45L and K87L/K118L) of mVAPB and measured their dissociation constants using ITC. While wild-type mVAPB showed a dissociation constant (*K*_d_) of 1.7 ± 0.2 μM for mMIGA2^pFFAT^, the binding affinities of K87L/K118L and K43L/K45L mutants were 3- and 5-fold lower than that of wild type mVAPB, respectively (Fig. 5f and Supplementary Table 2). This is consistent with previous data obtained using the phosphorylation mimic mutants of mMIGA2 (Fig. 5b, c), confirming again that phosphorylation of both serine residues in the MIGA2 FFAT motif is required for tight interaction with VAPB.

Next, we examined how anchoring of MIGA2 and VAPB in the membranes of mitochondria and the ER, respectively, affects lipid transfer. To mimic this condition, we used histidine tag fused mMIGA2^275–570^ (His-mMIGA2^275–570^) and mVAPB (His-mVAPB) and liposomes containing DGS-NTA(Ni) (Fig. 5g). His-mVAPB was physically immobilized with donor liposomes comprising both Rhod-PE and NBD-PS. The donor liposomes pre-anchored with His-mVAPB were then mixed with acceptor liposomes pre-anchored with His-mMIGA2^275–570^ (Fig. 5g), after unbound His-mMIGA2^275–570^ and His-mVAPB had been removed by centrifugation. First, we measured dynamic light scattering (DLS) to test whether the S292E/S295D mutant actually induced the formation of a liposome cluster, and found that unlike wild-type His-mMIGA2^275–570^, liposome particle size was increased by approximately 5-fold in the presence of the His-mMIGA2^275–570^ (S292E/S295D) phosphorylation mimic mutant (Fig. 5h). No particle size increases were observed with histag-free mMIGA2^275–570^ or mMIGA2^275–570^ (S292E/S295D) (Fig. 5h), indicating that the liposome cluster was generated by the interaction between phosphorylation mimic mutation of MIGA2 FFAT motif and VAPB, when both were tethered to liposomes. Notably, the His-mMIGA2^275–570^ (S292E/S295D) mutant showed a roughly 4-fold higher transfer rate than that of wild-type His-mMIGA2^275–570^ (Fig. 5i and Supplementary Fig. 7c). The Histag-free mMIGA2^275–570^ or mMIGA2^275–570^ (S292E/S295D) mutant did not show a difference in transfer rate (Supplementary Fig. 9c). The result suggests that the phosphorylation-induced MIGA2-VAPB interaction enhances the lipid transfer rate when both proteins are tethered.

### MIGA2 associates with membranes through a positively charged surface

**Fig. 6:**
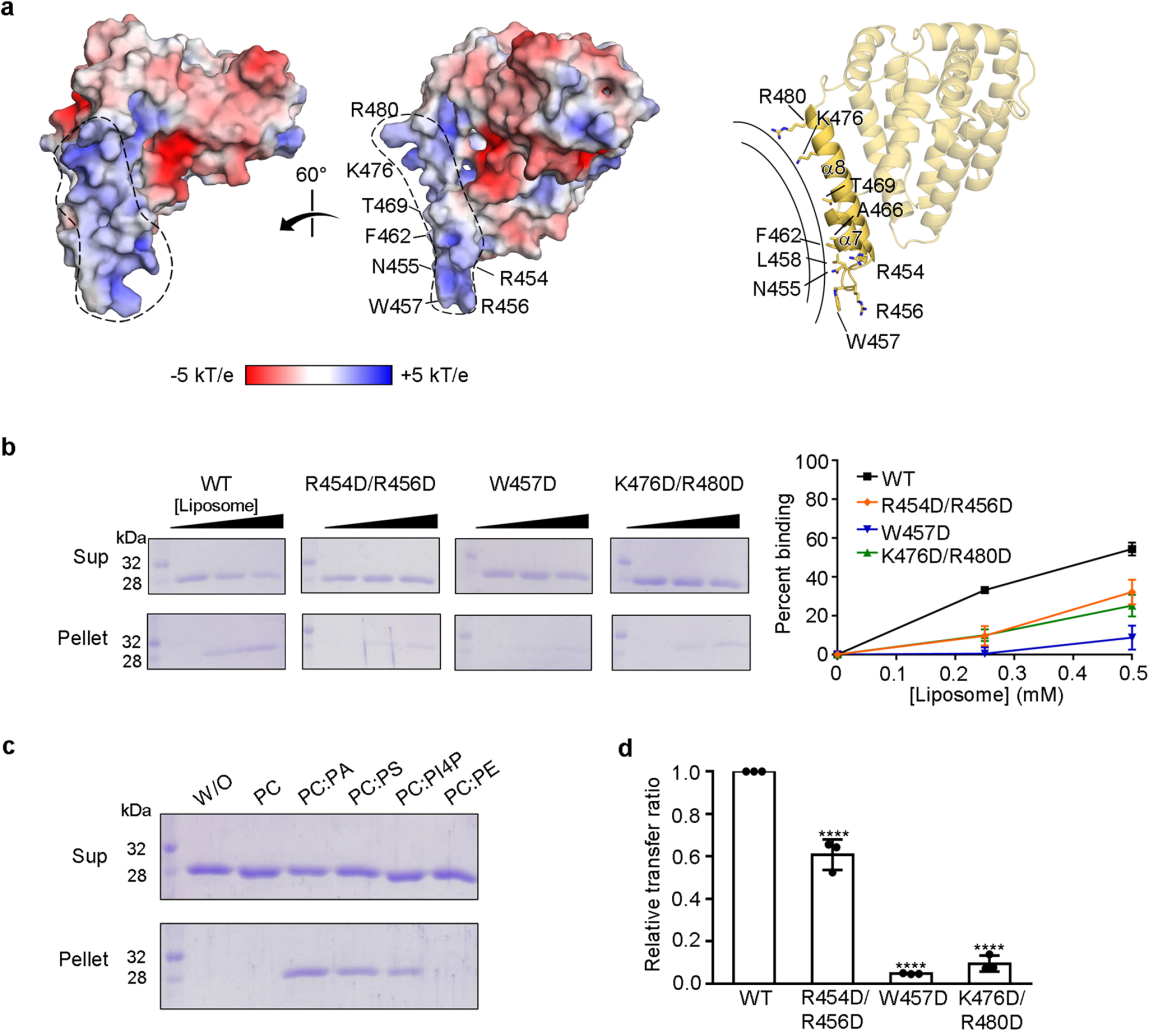
zMiga2 docks with the membrane through a positively charged concave surface. **a** Molecular surface of the zMiga2, colored according to electrostatic potential. The two views are 60° rotation around a vertical axis. The electrostatic potential was calculated with the APBS program^57^ and colored from −5 (red) to +5 (blue) kT/e (k, Boltzmann’s constant; T, temperature; e, charge of an electron). The membrane-associated region of zMiga2 is highlighted by dashed lines. A ribbon diagram on the right panel (in the same orientation as the middle panel) shows key residues involved in the association with the membrane. **b** Liposome sedimentation assay showing the direct membrane association of zMiga2. The experiments using wild type and mutants (R454D/R456D, W457D, and K476D/R480D) of zMiga2 were performed as shown in Fig. 1c. The right panel shows the quantification data using left (*N* = 3 independent experiments; dot shows mean ± SD). **c** Liposome sedimentation assay using a series of liposomes (PC, PC:PA, PC:PS, PC:PI4P, and PC:PE) (*N* = 2 independent experiments). **d** A graph comparing the transfer ratio between zMiga2 WT and each mutant (R454D/R456D, W457D, and K476D/R480D). Lipid transfer activity was compared with that of WT using one-way ANOVA (*N* = 3 independent experiments; individual data point shown as dots, bars show mean ± SD). ****p < 0.0001 (R454D/R456D, W457D, and K476D/R480D). Source data are provided in a Source data file.

Lastly, we investigated which region of MIGA2 physically associates with membranes to transfer phospholipids. Based on its structure, we found that zMiga2 has a positively charged concave surface in the handle region composed of helices α7 and α8 (Fig. 6a). These helices protrude from the helical core. Contributing to the concave surface are the solvent-exposed side chains of K476 and R480 from helix α8 as well as the side chains of W457, R454, and R456 from the loop between helices α7 and α8 (Fig. 6a). Sequence analysis confirmed that the residues of this region are highly conserved among other ortholog species (Supplementary Figs. 3 and 4b). We imagined that this positively charged concave region might complement a negatively-charged convex membrane surface. Liposome binding experiments with mutant proteins showed that R454D/R456D and K476D/R480D double mutants as well as the W457D single point mutant had reduced membrane-binding activity, suggesting that zMiga2 could indeed contact the membrane through its positively charged concave surface formed by the α7 and α8 helices of the handle (Fig. 6b). We also confirmed that there were no folding defects in these mutants (Supplementary Fig. 6a).

Next, we examined which type of phospholipid MIGA2 favored for membrane binding. To this end, we prepared a series of liposomes consisting of various combinations of PC-based glycerophospholipids, and carried out liposome binding experiments. As shown in Fig. 6c, MIGA2 specifically bound to liposomes containing PA, PS, and PI4P but not PC and PE.

Lastly, we examined whether the membrane contact of zMiga2 via helices α7 and α8 would affect lipid transfer. The lipid transfer assay showed that the R454D/R456D mutation reduced the rate of lipid transfer to 60% of that of wild-type zMiga2 (Fig. 6d and Supplementary Fig. 7d). The W457D or K476D/R480D mutations reduced the lipid transfer ability to 15% (Fig. 6d and Supplementary Fig. 7d). We conclude that the docking of zMiga2 to the membrane through helices α7 and α8 helices is required for lipid trafficking.

Previously, it has been proposed that MIGA2 plays a role in de novo lipogenesis by linking mitochondria, the ER, and LDs^28^. Their analyses using truncated constructs revealed that the amphipathic segment (residues 450–550, equivalent to residues 447–547 in zMiga2) in MIGA2 mediates the MIGA2 interaction with LDs. Based on our structure, we propose that the amphipathic segment in their study corresponds to helices α9 and α10, which we suggest form part of the hydrophobic cavity required for lipid binding. However, the α10 helix contacts helices α7 and α8, which we suggested from the membrane docking region. Any disruption of this contact might affect the stability of this region and impair the membrane docking of MIGA2, which is not inconsistent with this previous data^28^.

## Discussion

**Fig. 7:**
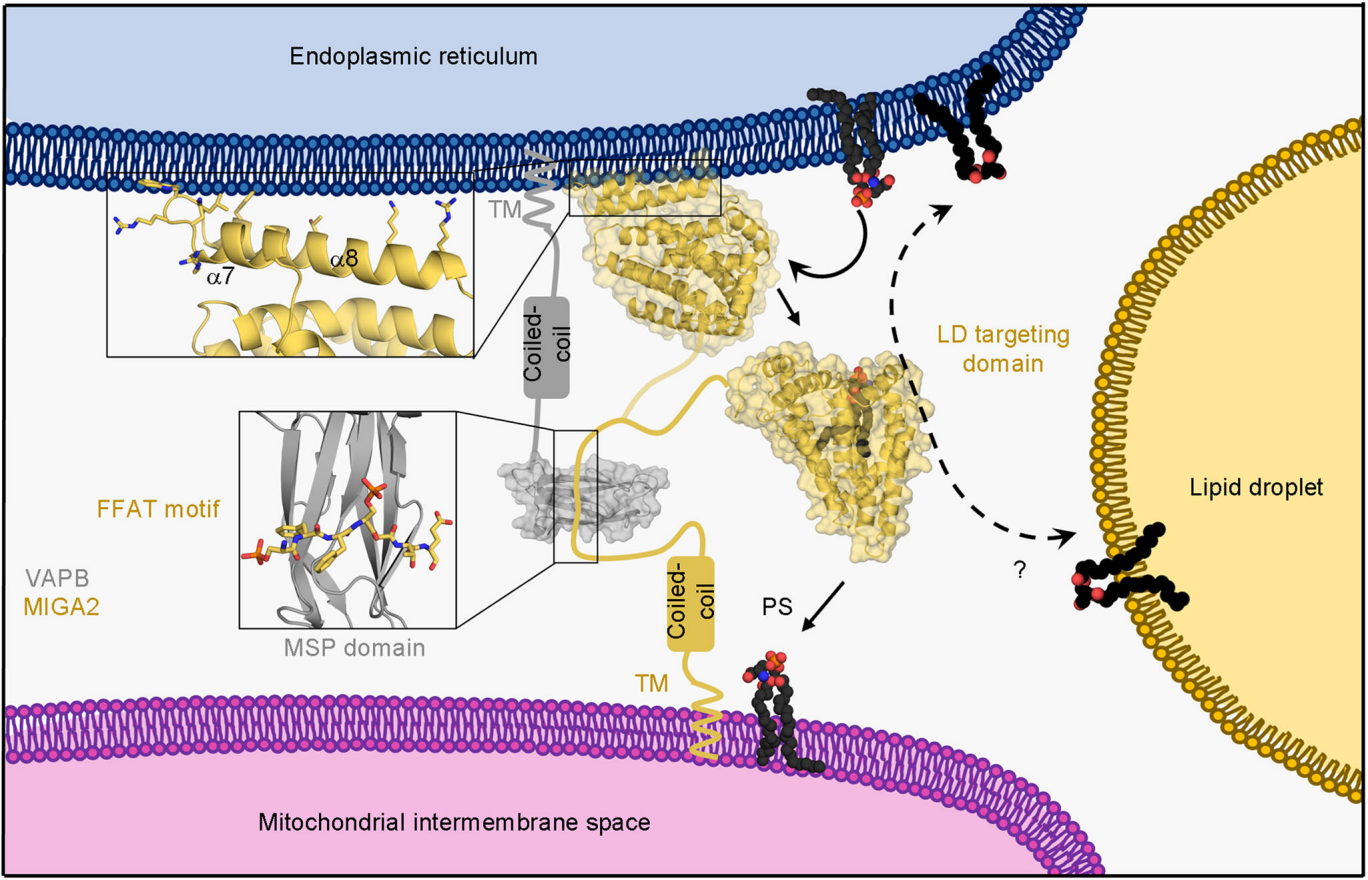
Putative working model of MIGA2 at the ERMCS. Based on the results of this study, we propose a putative lipid trafficking mechanism for MIGA2 between ER (blue), mitochondria (pink), and lipid droplet (yellow) membrane contact sites. MIGA2 (yellow orange) forms a complex with VAPB (gray) via the phosphorylated FFAT motif and then transfers lipids between the membranes by extracting and interiorizing lipids into its large hydrophobic cavity.

In this study, we have established the molecular mechanism via which MIGA2 traffics glycerophospholipids between membranes of the ER and mitochondria (Fig. 7). The crystal structure of zMiga2 revealed a hydrophobic cavity for lipid binding, and biochemical experiments demonstrated that MIGA2 can transfer various phospholipids between liposomes. Structural similarity among zMiga2, APOE, and TIP47 confirmed the MIGA2 was evolutionary designed to be a lipid-binding protein (Fig. 2d and Supplementary Fig. 5)^36–38^. Although zMiga2 shares the spatial arrangement of secondary structures with APOE and TIP47, it is only zMiga2 that contains a large hydrophobic cavity. Structure studies of APOE and TIP47 suggested that these proteins interact with lipids via a hydrophobic surface rather than via a cavity^36–38^. We suggest that the unusual cavity structure of MIGA2 would make it more efficient in lipid transport.

It is interesting that MIGA2 has a higher preference for binding and transfer of PS than other lipids. Our lipid transfer assay showed that MIGA2 can specifically transfer PS 6~12 times faster than PC, PA, PE, or CER (Fig. 4e and Supplementary Fig. 7a). This is a surprising result because it was little known whether tethering proteins harbor phospholipid selectivity in the metazoan ERMCS. To date, no mammalian proteins involved in selectively transferring PS between the ER and the mitochondrion through a non-vesicular trafficking have been reported, except for ORP5. ORP5, an ER membrane protein, was originally found to transfer PS and PI4P between the ER and PM via its pleckstrin homology (PH) domain, which associates with PM^24^. A recent study suggested that ORP5 is also recruited to mitochondria via its association with PTPIP51 or the sorting and assembly machinery component 50 homolog (SAM50) of the MIB complex, and may be involved in transferring PS between the ER and mitochondria^23,46^. Another study has revealed that the cytosolic synaptic vesicle membrane protein VAT-1 homolog (VAT1) might be involved in phospholipid trafficking at the ER-mitochondria contact region^47,48^. Yet, there is no direct evidence showing how VAT1 targets the ER or mitochondria. A structural and biochemical study of the TPR domain of PTPIP51 showed that it could bind phospholipids through its cavity and prefers to transfer PA, in vitro^22^. However, our lipid transfer assay clearly showed that, compared with Osh6, a yeast homolog of ORP5/8, MIGA2 prefers to transfer PS (Fig. 4f and Supplementary Fig. 7b), indicating that MIGA2 largely contributes to PS transport in the ERMCS in mammalian cells^24,40–42^. Also, whereas scOsh6 is limited to transferring substrates other than PS or PI4P (Fig. 4f and Supplementary Fig. 7b, e)^40,41^, zMiga2 transfers not only PS, but also PE, PC, and PA, despite showing a relatively low transfer rate (Fig. 4e and Supplementary Fig. 7d). Based on these results, we propose that MIGA2 plays a key role in transporting pan-phospholipids at the ERMCS. Likewise, in yeast, the ERMES complex transfers a wide spectrum of phospholipids substrates between the ER and mitochondria, although it has no obvious selectivity toward certain phospholipids^11–15^. Since the conversion of PS to PE occurs in mitochondria and PE is a substrate for generating PC, the transport of phospholipids between ER and mitochondria would be essential for maintaining phospholipids homeostasis in cells^6,49^. Because non-vesicular phospholipid trafficking at ER-mitochondrial MCSs might be the predominant mechanism and important for rapid trafficking, we believe that the redundant pathway through MIGA2 could assist in efficient phospholipid trafficking at this MCS^6,49^.

How does MIGA2 preferentially transport PS among pools of diverse glycerophospholipids? Based on our structure, we postulate that the two perpendicular helices (α2 and α5) in the lid structure of zMiga2 might be involved in selecting specific phospholipids. These helices enclose the head group of the bound phospholipid (Supplementary Fig. 10a, b). In particular, side chains of the highly conserved L361 of α2, and W409 and L416 of α5 project toward the head group, although they do not make direct contact with the phospholipid. To prove this hypothesis we would have liked to solve the structure of PS bound to MIGA2, but unfortunately we have been unable to grow diffraction-quality crystals. However, we have indirect evidence to show that the W409A mutant has an approximately 2-fold higher NBD-PE binding and transfer rate than the wild type, in which the NBD moiety is conjugated at the head group of the phospholipid (Supplementary Fig. 10c–e). There was no difference in transfer rate when the NBD moiety was conjugated on the hydrocarbon tail of the phospholipid (Supplementary Fig. 10d, e). Mutation of tryptophan to alanine might reduce steric hindrance and generate a relatively large space at this region, resulting in phospholipids with bulky headgroup being easily picked up by zMiga2. Nevertheless, since this mutant exhibited no changes in lipid selectivity, further work is needed to address this question.

Lastly, we have shown that the interaction between MIGA2 and VAPB accelerates lipid transfer when they are anchored in the membrane (Fig. 5i). Furthermore, we provide biochemical evidence showing that the MIGA2-VAPB interaction could be regulated by phosphorylation (Fig. 5a–f)^29^. In fact, the FFAT motif in MIGA2 does not have the consensus sequence observed in other FFAT motifs such as those in ORP1 and NIR2^43,44^. Rather, some acidic residues of the conventional FFAT motif are substituted with conserved serine residues in MIGA2 FFAT, raising the possibility that the MIGA2 FFAT motif could be modified by phosphorylation. In this study, we showed that phosphorylations at both S292 and S295 of mouse MIGA2 FFAT motif are required for its tight interaction with the VAPB using structural and biochemical analyses (Fig. 5a–f and Supplementary Fig. 9a, b). The intermolecular interactions generated by phosphorylation assist not only the formation of MCSs but also facilitate rapid lipid trafficking (Fig. 5 and Supplementary Figs. 7c and 9). Taken together, our study provides evidence that the formation of the ERMCS and the resultant lipid synthesis could be regulated by protein phosphorylation.

In conclusion, the structural information and biochemical experiments reported in this paper provide a framework to understand the molecular mechanism by which MIGA2 mediates the formation of ER-mitochondria contact sites and facilitates lipid trafficking between anchored membranes in mammalian cells.

## Methods

### Cloning, protein expression, and purification

For protein preparation, the *Mus musculus* and *Danio rerio* MIGA2 and *Mus musculus* MIGA1 sequences were cloned into the modified pETDuet-1 vector containing a TEV cleavage site. Mouse VAPB and *S. cerevisiae* Osh6 sequences were cloned into pGEX-6P-1 and pET28b-SMT3 vectors, respectively. The proteins were expressed in *Escherichia coli* strain BL21 (DE3). Bacteria were grown in Luria-Bertani medium at 37 °C to OD_600_ of 0.7 and induced by 0.4 mM isopropyl β-_D_-1-thiogalactopyranoside at 18 °C for 18 h. MIGA and scOsh6 proteins were purified by Ni^2+^-chelated HiTrap column chromatography (GE Healthcare, USA) and mVAPB was purified by GST affinity chromatography. The His x6, His x6-SUMO, and GST tag were cleaved by TEV, Ulp1, and PreScission protease at a ratio of 1:50, 1:500, and 1:200 (w/v), respectively. Proteins were further purified by size exclusion chromatography (Superdex 200 column, GE Healthcare) in buffer A (25 mM Tris-HCl pH 7.5, 150 mM NaCl, and 5 mM DTT). For selenomethionine-derivatized protein, the zMiga2 plasmid was transformed and expressed in B834 (DE3) grown in M9 minimal media containing selenomethionine. All mutants were generated by PCR-based mutagenesis and confirmed with DNA sequencing. All mutants were purified as described above.

For mVAPB-mMIGA2^pFFAT^, the phosphorylated FFAT motif peptide corresponding to *M. musculus* MIGA2 residues 289–298 (sequence SEDpSFFpSATE) was synthesized with N-terminal acetylation and C-terminal amidation, and purified by HPLC. The purified mVAPB was mixed with the peptide at a 1:2 molar ratio at 4 °C overnight.

### Crystallization and structure determination

zMiga2 was crystallized by the hanging-drop vapor diffusion method in reservoir buffer comprising 18% (w/v) SOKALAN cp5, 100 mM HEPES pH 7.0, and 300 mM ammonium formate at 18 °C. The crystal was harvested into cryo-solution containing 30% (v/v) glycerol and flash-frozen in liquid nitrogen. X-ray diffraction data were collected on the 5C beamline of the Pohang Accelerator Laboratory and processed with the program HKL2000^50^. Phases were calculated from a SAD dataset using the PHENIX program^51^. Structures were built using Coot^52^ and refined with PHENIX. The final model was refined to R/R_free_ values of 0.1997/0.2390.

The mVAPB-mMIGA2^pFFAT^ complex was concentrated to 17 mg/ml and crystallized by the hanging-drop method at 18 °C by mixing 1 μl of protein solution with 1 μl of well solution comprising 1.8 M ammonium sulfate, 100 mM phosphate-citrate pH 4.2, and 2% (v/v) iso-propanol. Crystals were transferred to a solution comprising well solution plus 30% (v/v) glycerol and flash-frozen in liquid nitrogen. Diffraction data were collected at beamline 7 A of the Pohang Accelerator Laboratory at an X-ray wavelength of 0.9793 Å. The crystal structure was determined by molecular replacement with the program phaser (PHENIX), using the coordinates of VAPB (PDB ID: 3IKK) as a search model^53–55^. The final model was refined to R/R_free_ values of 0.1864/0.2281 with 1.68 Å native data. The X-ray data and refinement statistics are summarized in Supplementary Table 1.

### Lipid-binding assay

For the lipid-binding assays, 19 μl of 0.5 mg/ml wild-type MIGA2 or one of its mutants and MIGA1 in buffer A was mixed with 1 μl of 1 mg/ml NBD-PE (1,2-dioleoyl-sn-glycero-3-phosphoethanolamine-N-(7-nitro-2-1,3-benzoxadiazol-4-yl), Avanti Polar Lipids, USA) on ice. After 2 h, reaction products were subjected to CN-PAGE^13,14^. The reaction products were detected by fluorescence (ImageQuant LAS 4000, GE Healthcare) followed by Coomassie blue staining. Signal intensities were quantified with ImageJ software, and data were analyzed with GraphPad Prism 7.

### Lipid displacement assay

For lipid displacement assays, C‐terminal GST‐tag fused zMiga2 (zMiga2-GST) was incubated with a two-fold molar ratio of 16:0-12:0 NBD-PC (1-palmitoyl-2-{12-[(7-nitro-2-1,3-benzoxadiazol-4-yl)amino]dodecanoyl}-sn-glycero-3-phosphocholine, Avanti Polar Lipids) on ice. After 1.5 h, to remove unbound NBD‐PC, zMiga2-GST was incubated with Glutathione Sepharose 4B (GE Healthcare) beads for 30 min at 4 °C. Then, the reaction products were washed five times with buffer A containing 0.2% Nonidet P-40 (Sigma Aldrich, USA). NBD-PC bound zMiga2-GST was eluted in buffer A with 10 mM reduced glutathione and concentrated to 0.5 mg/ml. The mixture (20 μl) was incubated with the indicated concentrations of phospholipids and cholesterol on ice. After 2 h, products were subjected to 11% CN-PAGE^13,14^. All data were analyzed in the same way as in the lipid-binding assay.

### Liposome preparation

Lipids dissolved in chloroform were mixed at the desired molar ratio, and the solvent was evaporated by nitrogen gas. The lipid was hydrated with buffer B (20 mM HEPES pH 7.0 and 150 mM NaCl). After five freeze and thaw cycles with liquid nitrogen, liposomes were extruded through a mini-extruder (Avanti Polar Lipids) with 100 nm polycarbonate filter^56^.

All lipids used in this study were purchased from Avanti Polar Lipids: PS (1,2-dioleoyl-sn-glycero-3-phospho-L-serine), PC (1,2-dioleoylsn-glycero-3-phosphocholine), PE (1,2-dioleoyl-sn-glycero-3-phosphoethanolamine), PA (1,2-dioleoyl-sn-glycero-3-phosphate), PI3P (1,2-dioleoyl-sn-glycero-3-phospho-(1′-myo-inositol-3′-phosphate)), PI4P ((1,2-dioleoyl-sn-glycero-3-phospho-(1′-myo-inositol-4′-phosphate)), Cholesterol, NBD-PE, NBD-PS (1-palmitoyl-2-{12-[(7-nitro-2-1,3-benzoxadiazol-4-yl)amino]dodecanoyl}-sn-glycero-3-phosphoserine), NBD-PC, NBD-PA (1-palmitoyl-2-{12-[(7-nitro-2-1,3-benzoxadiazol-4-yl)amino]dodecanoyl}-sn-glycero-3-phosphate), NBD-Ceramide (N-[12-[(7-nitro-2-1,3-benzoxadiazol-4-yl)amino]dodecanoyl]-D-erythro-sphingosine), NBD-Cholesterol (5-cholesten-3ß-ol 6-[(7-nitro-2-1,3-benzoxadiazol-4-yl)amino]caproate), Rhod-PE (1,2-dioleoyl-sn-glycero-3-phosphoethanolamine-N-(lissamine rhodamine B sulfonyl)), and DGS-NTA(Ni) (1,2-dioleoyl-sn-glycero-3-[(N-(5-amino-1-carboxypentyl)iminodiacetic acid)succinyl]).

### Lipid extraction assay

For the mMIGA2 and mMIGA1^313–573^ lipid extraction assay, 15 μM of protein was mixed with 300 µM donor liposomes (NBD-PE:PC = 20:80) in 200 µl buffer B at room temperature. After 1 h, the mixture was loaded into a 1.5 ml ultracentrifuge tube (Beckman Coulter, USA) and mixed with 200 µl buffer B containing 80% (w/v) Nycodenz AG (Alere Technologies AS, Norway). Then, the mixture was overlaid with 400 µl buffer B containing 30% (w/v) Nycodenz AG, 300 µl buffer B containing 10% (w/v) Nycodenz AG, and then 200 µl buffer B. After centrifugation at 300,000 × *g* for 1 h, protein-containing fractions were analyzed for fluorescence (excitation wavelength, 460 nm; emission wavelength, 535 nm) using a spectrofluorometer (Tecan Infinite m200, Switzerland). Data were analyzed using GraphPad Prism 7.

### Lipid transfer assay

For measuring the lipid transfer activities of zMiga2, mMIGA2, mMIGA1^313–573^, and scOsh6, fluorescence resonance energy transfer (FRET) assay was used. At the indicated concentrations, equal concentrations of donor liposomes (PC:PE:NBD-lipid:Rhod-PE = 70:18:10:2) and acceptor liposomes (PC:PE = 80:20) were incubated in 100 µl buffer B at room temperature. Proteins were added to the liposome mixtures 3 min after the start of the assay^12,56^.

To measure the lipid transfer activity between His-mVAPB pre-anchored donor liposome and His-mMIGA2^275–570^ pre-anchored acceptor liposome, 26 µM of donor liposome (PC:PE:NBD-PS:Rhod-PE:DGS-NTA(Ni) = 66.5:21:10:2:0.5) was incubated with His-mVAPB, and an equal concentration of acceptor liposome (PC:PE:DGS-NTA(Ni) = 74.5:25:0.5) was incubated with His-mMIGA2^275–570^ (WT and S292E/S295D) at 4 °C for 1 h. Then, the liposomes were centrifuged at 300,000 × *g* for 1 h to remove unbound protein^12,30,56^, and the two samples were mixed together. NBD fluorescence was analyzed as described for the lipid extraction assay. Data were analyzed using GraphPad Prism 7 and Excel.

Lipid transfer rate was determined by [Substrate](*F*_t_ − *F*_c_)/{(*F*_max_ − *F*_c_)Δ*t*}. [Substrate] is the total substrate lipid concentration in the donor liposome. *F*_t_ is the intensity of NBD fluorescence at the indicated time *t*. *F*_c_ is the fluorescence intensity without protein. *F*_max_ is the total NBD fluorescence intensity of the donor liposome. *F*_max_ was measured after adding 0.1% Triton X-100. Δ*t* is the time difference between protein injection and the time point *t*^56^.

### Liposome-binding assay

For the liposome-binding assay, 20 μl of 5 mg/ml mMIGA1 or mMIGA2 or the wild type and mutants of zMiga2 were mixed with liposomes (PC:PE:PA:PS:PI3P:PI4P = 40:38:5:15:1:1) in 500 µl buffer B for 1 h at room temperature. Then, the mixture was centrifuged at 300,000 × *g* for 1 h and the supernatant and pellet fractions were subjected to 12% SDS-PAGE.

### GST pull-down assay

For the GST pull-down assay, C‐terminal GST-fused mVAPB was mixed with 5 μl of Glutathione Sepharose 4B beads in a 500 µl buffer A for 1 h at 4 °C. After incubation, beads were washed three times with buffer A. Subsequently, purified wild type or mutant mMIGA2^275–570^ was added to the mixture and incubated for 2 h at 4 °C. Then, beads were washed three times with buffer A containing 0.1% Triton X-100 and the proteins were subjected to 12% SDS-PAGE.

### Isothermal titration calorimetry (ITC) analysis

For ITC analysis, wild type and mutant mVAPB, mMIGA2^275–570^, and lyophilized mMIGA2^pFFAT^ were prepared in buffer C (25 mM Tris-HCl pH 7.5, 150 mM NaCl, and 4 mM β-mercaptoethanol). ITC measurements were carried out using a Microcal ITC200 instrument (Malvern Panalytical, UK) at 25 °C. Measurements were taken over 20 injections of 2 μl of sample with a reference power of 5 μcal/s and at a stirring speed of 1000 rpm. The data were fitted using the program Origin with a 1:1 binding model.

### Dynamic light scattering (DLS) analysis

DLS was measured using Zetasizer Nano ZS (Malvern Panalytical). To monitor the formation of liposome clusters mediated by MIGA2-VAPB interaction, 100 μM His-mVAPB pre-anchored donor liposomes (PC:PE:NBD-PS:Rhod-PE:DGS-NTA(Ni) = 66.5:21:10:2:0.5) were incubated with 100 μM His-mMIGA2^275–570^ (WT and S292E/S295D) pre-anchored acceptor liposomes (PC:PE:DGS-NTA(Ni) = 74.5:25:0.5) in buffer C for 30 min at 4 °C. In the control experiments, 100 μM liposome mixture (donor and acceptor liposomes) was incubated with mMIGA2^275–570^ (WT and S292E/S295D) and mVAPB in buffer C for 30 min at 4 °C. Samples were loaded into a glass cuvette (PCS1115, Malvern Panalytical) and light scattering was measured with 175° backscatter detection at 25 °C. Data were analyzed by Zetasizer software (Malvern Panalytical) and GraphPad Prism 7.

### Size exclusion chromatography (SEC) analysis

Analysis of the relative molecular masses of mMIGA2 proteins was carried out by size exclusion chromatography. Proteins were concentrated to 2–4 mg/ml and injected onto a Superdex 200 increase 10/300 GL column (GE Healthcare) equilibrated with buffer A. A molecular mass standard containing ferritin (440 kDa), aldolase (158 kDa), conalbumin (75 kDa), carbonic anhydrase (29 kDa), and ribonuclease A (13.7 kDa) was used for calibration.

### Circular dichroism (CD) spectroscopy

Structural changes of a wild-type and mutant zMiga2 (7 μM) were monitored using a circular dichroism (CD) spectrometer (Jasco J-815, UK) with wavelength scan method from 198 to 260 nm. All protein samples were prepared in 25 mM sodium phosphate pH 7.5, 150 mM sodium chloride. Data were analyzed by spectrum manager (Jasco Inc.) and GraphPad Prism 7.

Reagents used in this study are listed in Supplementary Table 3.

### Reporting summary

Further information on research design is available in the Nature Research Reporting Summary linked to this article.

## Supplementary information

Supplementary Information

Peer Review File

Reporting Summary

## Data Availability

The data that support this study are available from the corresponding authors upon reasonable request. The coordinates have been in the RCSB Protein Data Bank (PDB) under accession codes 7X15 (zMiga2) and 7X14 (mMIGA2^pFFAT^-mVAPB). The accession codes for 2L7B (APOE) and 1SZI (TIP47) were used. Source data are provided with this paper.

## Source data

Source data
