# Supplementary material for: Structural basis for mitoguardin-2 mediated lipid transport at ER-mitochondrial membrane contact sites
Source: Nat Commun. 2022 Jun 28;13:3702. doi: 10.1038/s41467-022-31462-6 (PMC9239997; doi:10.1038/s41467-022-31462-6)
Supplement: Supplementary file 1 — Supplementary Information [file 41467_2022_31462_MOESM1_ESM.pdf]

Supplementary Information

Structural basis for mitoguardin-2 mediated lipid transport at ER-mitochondrial membrane contact sites

Hyunwoo Kim[1,3], Seowhang Lee[1], Youngsoo Jun[2,3] and Changwook Lee[1,3]*

[1]Department of Biological Sciences, Ulsan National Institute of Science and Technology, 50 UNIST-gil, Ulsan 44919, Korea.

[2]School of Life Sciences and [3]Cell Logistics Research Center, Gwangju Institute of Science and Technology, Gwangju 61005, Korea.

*Correspondence regarding this manuscript to:

Changwook Lee, Ph.D.

Department of Biological Sciences,

Ulsan National Institute of Science and Technology (UNIST)

50 UNIST-gil, Ulsan 44919, Korea.

Telephone – 82-52-217-2534; Fax – 82-52-217-2639

E-mail – changwook@unist.ac.kr

Supplementary Figures

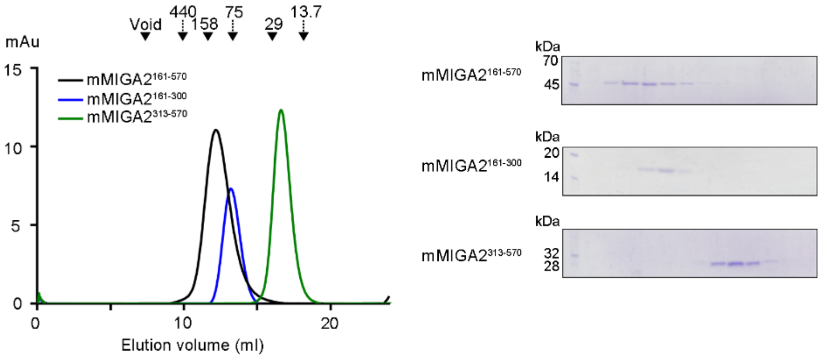

Supplementary Fig. 1. Size exclusion chromatography (SEC) analysis of mMIGA2. mMIGA2 proteins (mMIGA2$^{161–570}$, mMIGA2$^{161–300}$, and mMIGA2$^{313–570}$) were loaded onto a Superdex 200 increase 10/300 GL column. mMIGA2$^{313–570}$ forms a monomer. However, other MIGA2 constructs containing the middle domain (residues 161–300) eluted from the column earlier than their calculated molecular weights, indicating that the MIGA2 middle domain forms an oligomer. The standard molecular masses are shown above the chromatogram curves to allow comparison of the relative molecular masses (ferritin, 440 kDa; aldolase, 158 kDa; conalbumin, 75 kDa; carbonic anhydrase, 29 kDa; ribonuclease A, 13.7 kDa) (left). SDS-PAGE of the eluted proteins is shown on the right (N = 2 independent experiments).

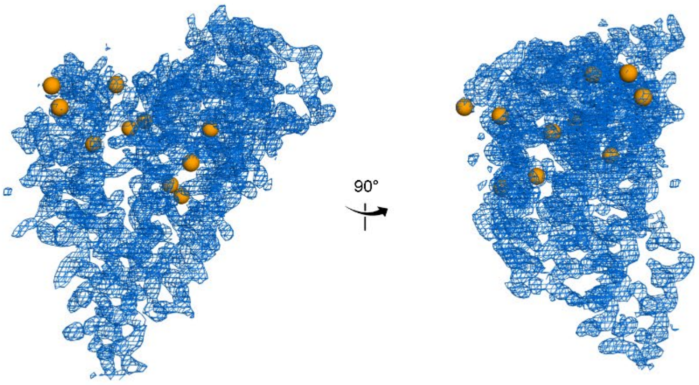

Supplementary Fig. 2. Structural analysis of zMiga2. Experimental electron density map (calculated with data to 2.85 Å resolution and contoured at 1.0 σ) of the crystal asymmetric unit of the zMiga2. The map was calculated with Se-SAD phases after density modification. Orange spheres represent selenium atoms.

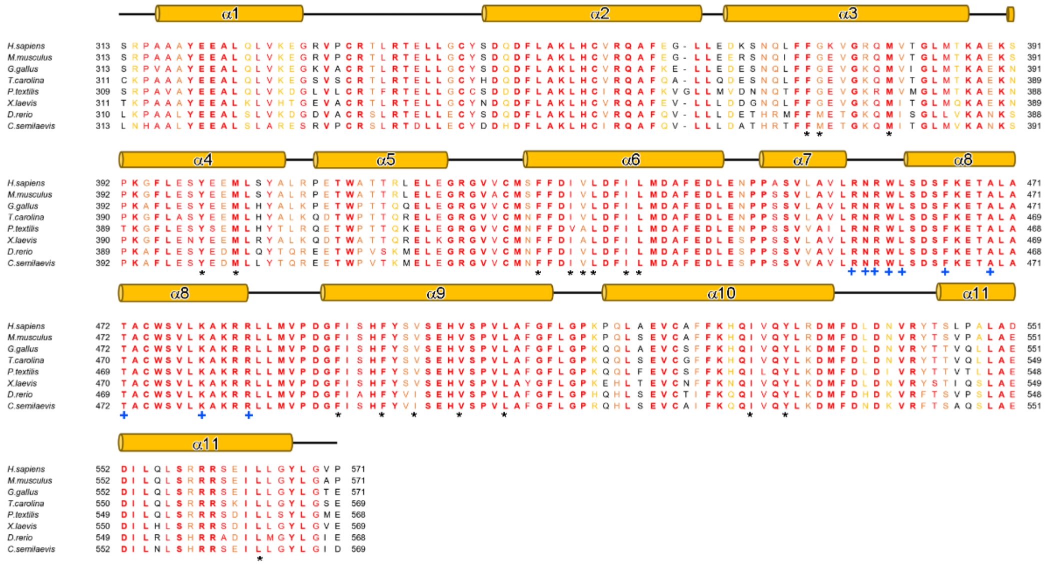

Supplementary Fig. 3. Sequence alignment of conservation of MIGA2 LD targeting domain from eight species. Sequence alignment of MIGA2 LD targeting domain from *Homo sapiens* (UniProt entry: Q7L4E1), *Mus musculus* (UniProt entry: Q8BK03), *Gallus gallus* (A0A1D5PFL2), *Terrapene carolina* (A0A674KEF0), *Pseudonaja textilis* (A0A670ZBH5), *Xenopus laevis* (Q6GR21), *Danio rerio* (Q5BLE2), and *Cynoglossus semilaevis* (A0A3P8WGZ8). Secondary structure elements, based on the crystal structure, are shown above the sequences, with α helices depicted as cylinders and loops as black solid lines. The sequence conservation at each amino acid is represented with a color gradient from yellow (70% similarity) to bold red (100% identity). Black star and blue plus marks below the sequences indicate residues involved in the lipid binding and membrane binding, respectively.

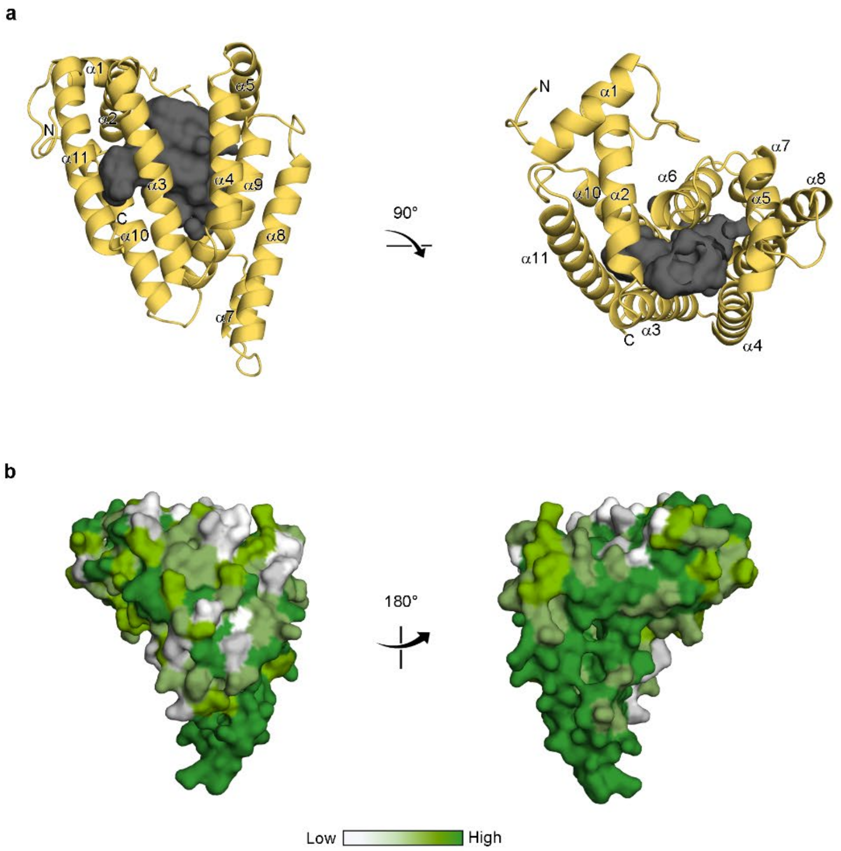

Supplementary Fig. 4. Overall structure of zMiga2 showing lipid binding cavity and surface conservation. a Pictures show the overall structure of zMiga2 (yellow orange). The central hydrophobic cavity (black) for lipid binding was calculated using CASTp 3.0 and a 1.2 Å probe radius[1]. **b** Surface representation of the zMiga2 molecule. Residue conservation was analyzed by Consurf[2] server (consurf.tau.ac.il) using 150 different organism orthologs and colored according to conservation from low (white) to high (green) identity.

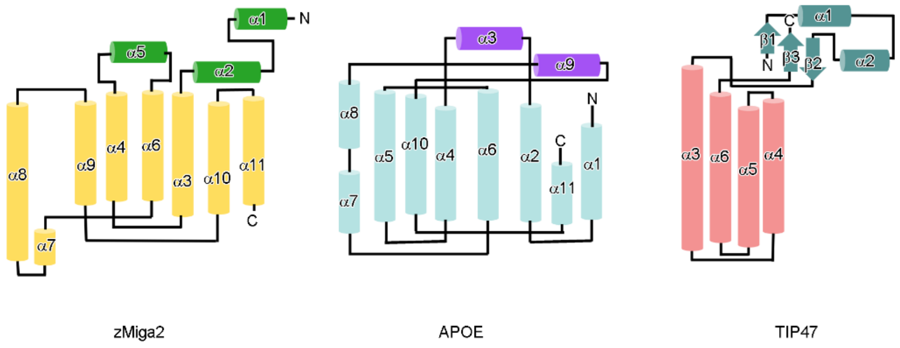

Supplementary Fig. 5. Schematic diagram of the structures of zMiga2, APOE, and TIP47. Schematic diagram shows the secondary structural elements and their organization in zMiga2 (lid: green; helix bundle: yellow orange), APOE (lid: purple; helix bundle: cyan), and TIP47 (lid: teal; helix bundle: pink). The α-helices and β-strands are represented by cylinders and arrows, respectively.

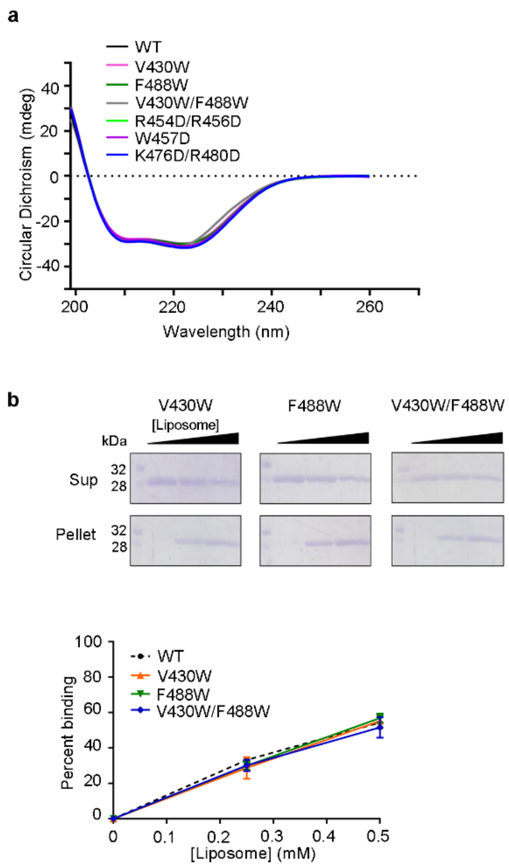

Supplementary Fig. 6. zMiga2 mutants do not show structural changes. a The figure shows the far-UV CD spectra scan from 198 to 260 nm for wild type and mutant (V430W, F488W, V430W/F488W, R454D/R456D, W457D, and K476D/R480D) zMIGA2. **b** Liposome sedimentation assay using zMiga2 (V430W, F488W, and V430W/F488W). The experiment was performed as shown in Fig. 1c. Supernatants (sup) and pellets were analyzed by 12% SDS-PAGE and Coomassie blue staining (top). Quantification data (N = 3 independent experiments; dot shows mean ± SD) are shown below.

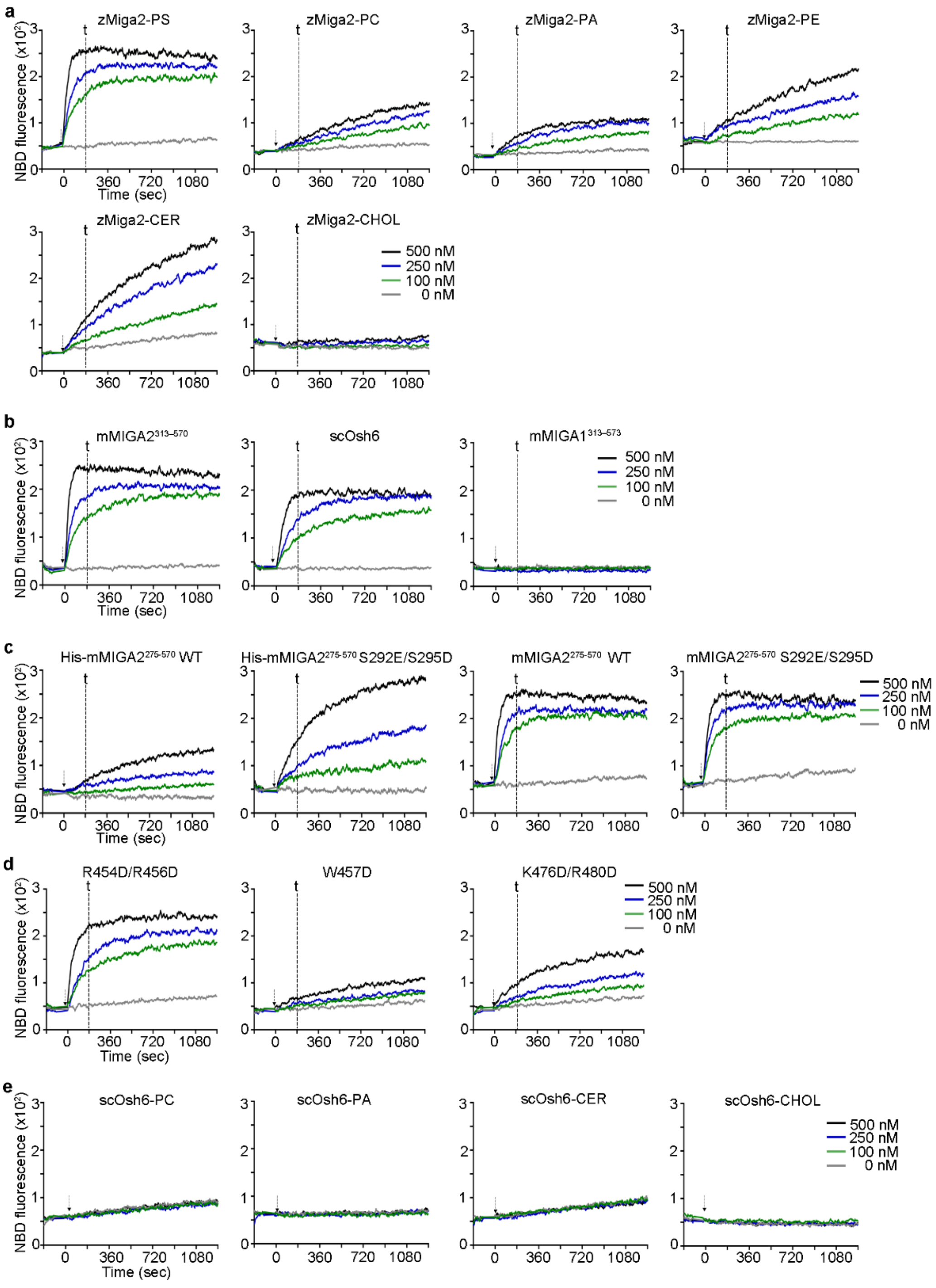

Supplementary Fig. 7. *In vitro* lipid transfer assay of MIGA2, MIGA1, and Osh6. Lipid transfer activity was assessed by FRET using liposomes (26 µM) at 25°C. The figures shown here are the raw data for Figs. 4e, f, 5i, and 6d quantification data in the main text. See the Methods section in the main text for the detailed experimental procedure. Curves colors represent the

indicated protein concentrations. The arrows indicate the starting point of measurement after protein injection and the time point “t” indicates the time used to calculate the lipid transfer rate. **a** Lipid transfer assay of zMiga2 against substrate ligands of PS, PC, PA, PE, CER, and CHOL. Donor liposomes comprise PC:PE:NBD-PS:Rhod-PE for PS; PC:PE:NBD-PC:Rhod-PE for PC; PC:PE:NBD-PA:Rhod-PE for PA; PC:PE:NBD-PE:Rhod-PE for PE; PC:PE:NBD-CER:Rhod-PE for CER; and PC:PE:NBD-CHOL:Rhod-PE for CHOL. **b** Lipid transfer assay of mMIGA2$^{313–570}$, scOsh6, and mMIGA1$^{313–573}$ for the PS substrate. **c** Lipid transfer assay of mMIGA2$^{275–570}$-mVAPB complex for the PS substrate. **d** Lipid transfer assay of zMiga2 R454D/R456D, W457D, and K476D/R480D for the PS substrate. **e** Lipid transfer assay of scOsh6 against substrate ligands of PC, PA, CER, and CHOL. Donor liposome composition is the same as for (**a**).

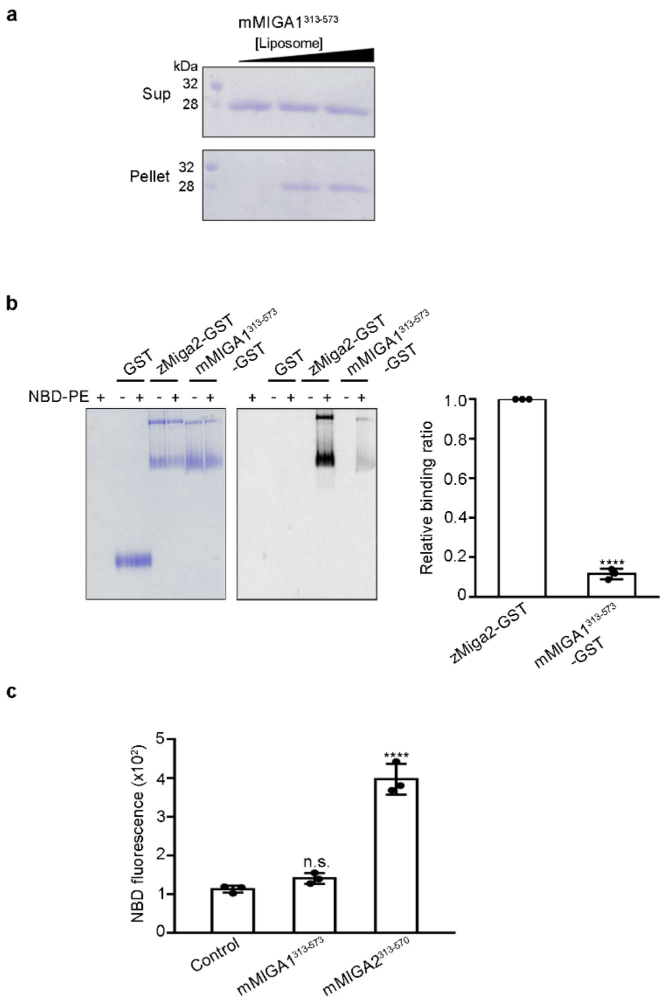

Supplementary Fig. 8. MIGA1 was unable to extract glycerophospholipid. a Liposome sedimentation assay using mMIGA1$^{313–573}$. Supernatants (sup) and pellets were analyzed by 12% SDS-PAGE and Coomassie blue staining (N = 2 independent experiments). **b** The lipid binding assay using purified zMiga2-GST and mMIGA1$^{313–573}$-GST. Proteins incubated with NBD-PE were subjected to 10% CN-PAGE and analyzed by Coomassie blue staining (left) and fluorescent detection (right). The bar graph shows quantification data. Lipid binding affinity of mMIGA1$^{313–573}$-GST was compared with that of zMiga2-GST using the two-sided t-test (N = 3 independent experiments; individual data point shown as dots, bar show mean ± SD, ****$p < 0.0001$). **c** Bar graph showing the results of lipid extraction by mMIGA1$^{313–573}$ and mMIGA2$^{313–570}$. Lipid extraction activity was compared with that of the control using one-way ANOVA (N = 3

independent experiments; individual data point shown as dots, bars show mean ± SD). ****$p < 0.0001$ (mMIGA2$^{313–570}$). Experiments in (**a**), (**b**), and (**c**) were carried out as shown in Fig. 1c, d, and f, respectively.

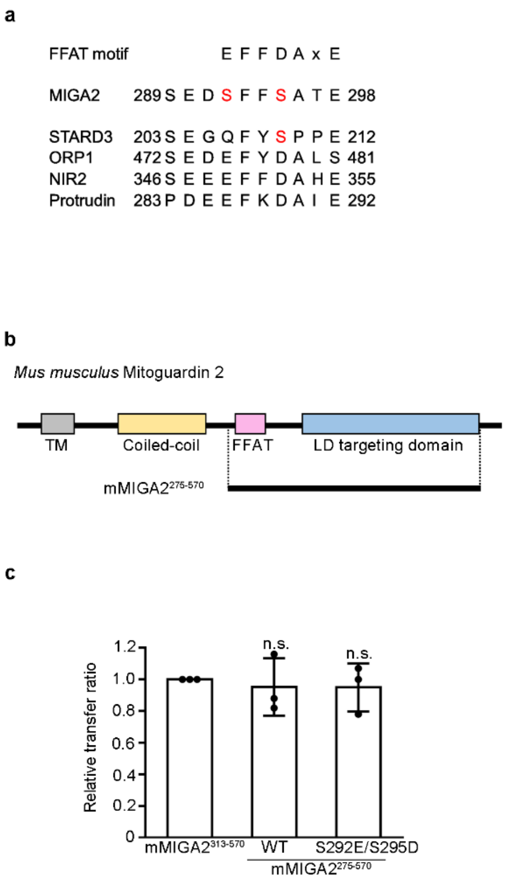

Supplementary Fig. 9. Interaction between the phosphorylated FFAT motif of MIGA2 and VAPB. a Sequence alignment of FFAT motifs from human MIGA2, STARD3, ORP1, NIR2, and Protrudin. Phosphorylation residues are indicated in red. **b** Domain organization of *Mus musculus* MIGA2. The mMIGA2$^{275–570}$ construct including the FFAT motif and LD targeting domain was used to examine the interaction. **c** The graphs show the transfer ratio when mMIGA2$^{275–570}$ (100 nM) and mVAPB (100 nM) were not associated with the liposome membrane. Liposome composition is the same as in Fig. 5h. The lipid transfer ratio was compared with that of mMIGA2$^{313–570}$ using one-way ANOVA (N = 3 independent experiments; individual data point shown as dots, bars show mean ± SD).

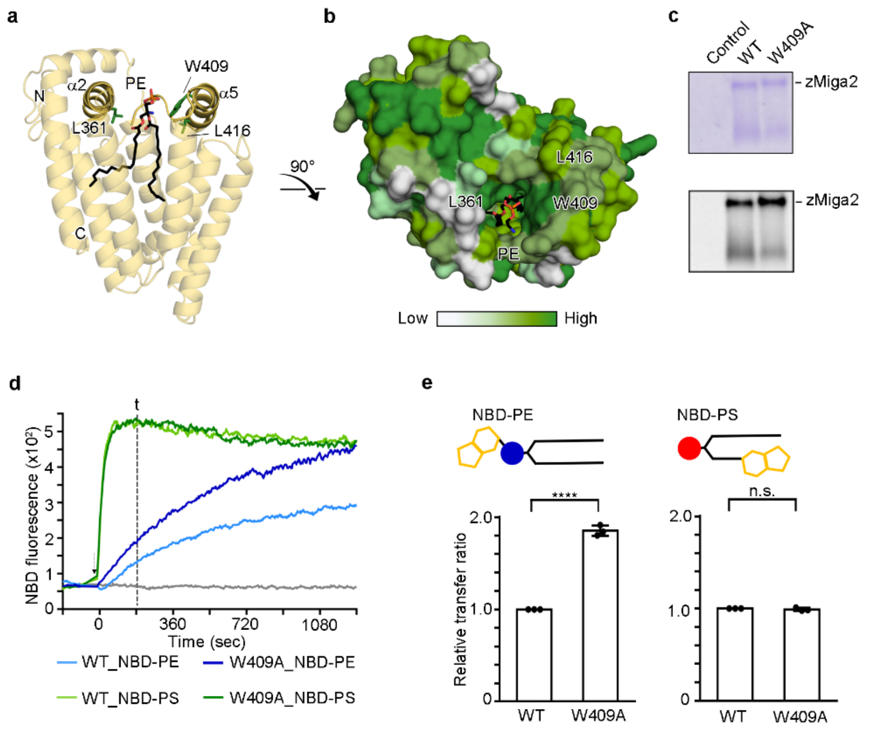

Supplementary Fig. 10. Possible model for PS selectivity of zMiga2. a Ribbon diagram and **b** surface conservation of zMiga2 highlighting the residues predicted to be involved in lipid selectivity. Two helices (α2 and α5) closely enclose the headgroup of bound phospholipids (PE). Color schemes are the same as in Fig. 3a and Supplementary Fig. 4b. **c** Lipid binding assay of zMiga2 WT and the W409A mutant (N = 3 independent experiments). Coomassie blue staining (top) and fluorescent detection (below). **d** *In vitro* lipid transfer assay of 2.5 mM zMiga2 WT and W409A was assessed by FRET at 25°C. Donor liposomes (50 µM) consist of PC:PE:NBD-PE:Rhod-PE or PC:PE:NBD-PS:Rhod-PE. **e** Schematic diagram of NBD-PE and NBD-PS structure showing the NBD conjugated site on phospholipids (top). The bar graphs show lipid transfer ratio between zMiga2 WT and W409A (below). Lipid transfer ratio of zMiga2 W409A was compared with that of zMiga2 WT using the two-sided t-test (N = 3 independent experiments; individual data point shown as dots, bar show mean ± SD, ****$p<0.0001$). The experiments from (**c**) to (**e**) were carried out as shown in Figs. 3b and 4c.

Supplementary Tables

Supplementary Table 1. Data collection and refinement statistics.

	zMiga2	mMIGA2^{pFFAT}-mVAPB
Dataset	Se-SAD	Native
X-ray source	Beamline 5C, PAL	Beamline 7A, PAL
Temperature (K)	100	100
Space group:	$P6_122$	$P2_12_12_1$
Cell parameters		
a, b, c (Å)	100.126, 100.126, 163.941	33.363, 44.507, 83.761
α, β, γ (°)	90.000, 90.000, 120.000	90.000, 90.000, 90.000
Data processing		
Wavelength (Å)	0.97942	0.97928
Resolution (Å)	50.00–2.85	50.00–1.68
CC1/2	0.997 (0.786)	0.995 (0.602)
I/σ	18.0 (1.58)	25.9 (4.93)
Completeness (%)	100.0 (100.0)	98.4 (100.0)
Redundancy	21.1 (18.4)	6.3 (5.2)
Measured reflections	453750	92537
Unique reflections	21478	14734
Refinement statistics		
Resolution (Å)	47.8800–2.8520	26.6950–1.6750
Reflections	21432	14685
Number of atoms		
Protein	2093	1049
Water	10	95
Ligand/ion	53	5
R-factor (%)	19.97	18.64
R_{free} (%)	23.90	22.81
RMSD		
Bond lengths (Å)	0.004	0.006
Bond angles (°)	0.744	0.912
Ramachandran plot, residues in		
Favored regions (%)	97.68	97.50
Allowed regions (%)	2.32	2.50
Disallowed regions (%)	0.00	0.00

*Highest resolution shell is shown in parenthesis.

Supplementary Table 2. ITC thermograms summarizing the measured thermodynamic parameters.

[Syringe] : [Cell]	K_d (μM)	N	ΔH (cal mol^{-1})	ΔS (cal mol^{-1} deg^{-1})
[mVAPB] : [mMIGA2$^{275\text{-}570}$ WT]	NB	-	-	-
[mVAPB] : [mMIGA2$^{275\text{-}570}$ S292E]	26.4 ± 6.8	0.633 ± 0.071	-6166 ± 884.9	0.255
[mVAPB] : [mMIGA2$^{275\text{-}570}$ S295D]	8.1 ± 1.5	0.501 ± 0.013	-1777 ± 268.1	-1.54
[mVAPB] : [mMIGA2$^{275\text{-}570}$ S292E/S295D]	2.5 ± 0.3	0.658 ± 0.009	-8999 ± 174.5	-4.58
[mVAPB WT] : [mMIGA2^{pFFAT}]	1.7 ± 0.2	0.755 ± 0.07	-14110 ± 179.8	-20.9
[mVAPB K43L/K45L] : [mMIGA2^{pFFAT}]	8.6 ± 1.4	1.04 ± 0.027	-11020 ± 432.0	-13.8
[mVAPB K87L/K118L] : [mMIGA2^{pFFAT}]	5 ± 0.4	0.698 ± 0.071	-12180 ± 419.2	-16.6

Supplementary Table 3. Materials information used in this study.

Name	Company	Cat. number
Nonidet P-40	Sigma Aldrich	21-3277
Nycodenz AG	Alere Technologies AS	1002424
SOKALAN cp5	BASF	30042761
DOPS (1,2-dioleoyl-sn-glycero-3-phospho-L-serine)	Avanti Polar Lipids	840035
DOPC (1,2-dioleoylsn-glycero-3-phosphocholine)	Avanti Polar Lipids	850375
DOPE (1,2-dioleoyl-sn-glycero-3-phosphoethanolamine)	Avanti Polar Lipids	850725
DOPA (1,2-dioleoyl-sn-glycero-3-phosphate)	Avanti Polar Lipids	840875
PI3P (1,2-dioleoyl-sn-glycero-3-phospho-(1'-myo-inositol-3'-phosphate))	Avanti Polar Lipids	850150
PI4P ((1,2-dioleoyl-sn-glycero-3-phospho-(1'-myo-inositol-4'-phosphate))	Avanti Polar Lipids	850151
Cholesterol	Avanti Polar Lipids	700100
NBD-PE (1,2-dioleoyl-sn-glycero-3-phosphoethanolamine-N-(7-nitro-2-1,3-benzoxadiazol-4-yl)	Avanti Polar Lipids	810145
NBD-PS (1-palmitoyl-2-{12-[(7-nitro-2-1,3-benzoxadiazol-4-yl)amino]dodecanoyl}-sn-glycero-3-phosphoserine)	Avanti Polar Lipids	810193
NBD-PC (1-palmitoyl-2-{12-[(7-nitro-2-1,3-benzoxadiazol-4-yl)amino]dodecanoyl})-sn-glycero-3-phosphocholine)	Avanti Polar Lipids	810131
NBD-PA (1-palmitoyl-2-{12-[(7-nitro-2-1,3-benzoxadiazol-4-yl)amino]dodecanoyl}-sn-glycero-3-phosphate)	Avanti Polar Lipids	810174
NBD-Ceramide (N-[12-[(7-nitro-2-1,3-benzoxadiazol-4-yl)amino]dodecanoyl]-D-erythro-sphingosine)	Avanti Polar Lipids	810211
NBD-Cholesterol (5-cholesten-3ß-ol 6-[(7-nitro-2-1,3-benzoxadiazol-4-yl)amino]caproate)	Avanti Polar Lipids	810251
Rhod-PE (1,2-dioleoyl-sn-glycero-3-phosphoethanolamine-N-(lissamine rhodamine B sulfonyl))	Avanti Polar Lipids	810150
DGS-NTA(Ni) (1,2-dioleoyl-sn-glycero-3-[(N-(5-amino-1-carboxypentyl)iminodiacetic acid)succinyl])	Avanti Polar Lipids	790404

Supplementary References

1 Tian, W., Chen, C., Lei, X., Zhao, J. & Liang, J. CASTp 3.0: computed atlas of surface topography of proteins. *Nucleic Acids Res* **46**, W363-w367, doi:10.1093/nar/gky473 (2018).

2 Ashkenazy, H. *et al.* ConSurf 2016: an improved methodology to estimate and visualize evolutionary conservation in macromolecules. *Nucleic Acids Research* **44**, W344-W350, doi:10.1093/nar/gkw408 (2016).
