# Supplementary material for: Structural basis for mitoguardin-2 mediated lipid transport at ER-mitochondrial membrane contact sites
Source: Nat Commun. 2022 Jun 28;13:3702. doi: 10.1038/s41467-022-31462-6 (PMC9239997; doi:10.1038/s41467-022-31462-6)
Supplement: Supplementary file 3 — Reporting Summary [file 41467_2022_31462_MOESM3_ESM.pdf]

nature portfolio

Corresponding author(s): Changwook Lee

Last updated by author(s): Jun 09, 2022

Reporting Summary

Nature Portfolio wishes to improve the reproducibility of the work that we publish. This form provides structure for consistency and transparency in reporting. For further information on Nature Portfolio policies, see our Editorial Policies and the Editorial Policy Checklist.

Statistics

For all statistical analyses, confirm that the following items are present in the figure legend, table legend, main text, or Methods section.

n/a	Confirmed	
☐	☒	The exact sample size (*n*) for each experimental group/condition, given as a discrete number and unit of measurement
☐	☒	A statement on whether measurements were taken from distinct samples or whether the same sample was measured repeatedly
☐	☒	The statistical test(s) used AND whether they are one- or two-sided<br>*Only common tests should be described solely by name; describe more complex techniques in the Methods section.*
☒	☐	A description of all covariates tested
☐	☒	A description of any assumptions or corrections, such as tests of normality and adjustment for multiple comparisons
☐	☒	A full description of the statistical parameters including central tendency (e.g. means) or other basic estimates (e.g. regression coefficient) AND variation (e.g. standard deviation) or associated estimates of uncertainty (e.g. confidence intervals)
☐	☒	For null hypothesis testing, the test statistic (e.g. *F*, *t*, *r*) with confidence intervals, effect sizes, degrees of freedom and *P* value noted<br>*Give P values as exact values whenever suitable.*
☒	☐	For Bayesian analysis, information on the choice of priors and Markov chain Monte Carlo settings
☒	☐	For hierarchical and complex designs, identification of the appropriate level for tests and full reporting of outcomes
☒	☐	Estimates of effect sizes (e.g. Cohen's *d*, Pearson's *r*), indicating how they were calculated

Our web collection on statistics for biologists contains articles on many of the points above.

Software and code

Policy information about availability of computer code

Data collection	The X-ray diffraction data were collected at the beamline 5C and 7A at the Phohang Accelerator Laberatory, Republic of Korea. FRET, DLS, ITC, and CD data were collected using the Tecan infinite 200, Zetasizer Nano ZS, MicrocalITC-200, and Jasco J-815, respectively.
Data analysis	X-ray diffraction data were processed with HKL2000, and structure was determined by using Phenix (ver 1.14), COOT (ver 0.8.9.2). Structure analysis using Consurf server, APBS Tool 2.1, and Dali server. FRET data was analyzed by Graphpad Prism 7 (ver 7.0) and Excel (ver 2205). ITC, CD, and DLS analysis using Origin 7 (ver 7.0552), Spectrum manager (ver 2.08.04), and zetasizer software (ver 7.1), respectively. Gel intensity was analyzed by Image J (ver 1.52a) and Graphpad Prism 7.

For manuscripts utilizing custom algorithms or software that are central to the research but not yet described in published literature, software must be made available to editors and reviewers. We strongly encourage code deposition in a community repository (e.g. GitHub). See the Nature Portfolio guidelines for submitting code & software for further information.

Data

Policy information about availability of data

All manuscripts must include a data availability statement. This statement should provide the following information, where applicable:

- Accession codes, unique identifiers, or web links for publicly available datasets
- A description of any restrictions on data availability
- For clinical datasets or third party data, please ensure that the statement adheres to our policy

The coordinates of the crystal structures have been deposited in the Protein Data Bank (PDB), with the accession codes 7X14 [https://doi.org/10.2210/pdb7X14/pdb] (mMIGA2pFFAT-mVAPB), 7X15 [https://doi.org/10.2210/pdb7X15/pdb] (zMiga2). The previously published structure of APOE and TIP47 used was obtained from the PDB: 2L7B [https://doi.org/10.2210/pdb2L7B/pdb] and 1SZI [https://doi.org/10.2210/pdb1SZI/pdb], respectively.

Field-specific reporting

Please select the one below that is the best fit for your research. If you are not sure, read the appropriate sections before making your selection.

- [x] Life sciences
- [ ] Behavioural & social sciences
- [ ] Ecological, evolutionary & environmental sciences

For a reference copy of the document with all sections, see nature.com/documents/nr-reporting-summary-flat.pdf

Life sciences study design

All studies must disclose on these points even when the disclosure is negative.

Sample size	All experiments were performed at least twice. Statistical test using one-way ANOVA (Figs. 1f, 3b, 4c, 4e, 4f, 5b, 6d and Supplementary Figs. 8c, 9c) and two-sided t-test (Fig. 5i and Supplementary Figs. 8b, 10e).
Data exclusions	No data was excluded.
Replication	All the presented experimental results were reliably reproduced, experiments were performed at least two times independently.
Randomization	For structure refinement, 5 % of reflection data were randomly chosen for calculating R-free value. For bio-chemical study, no randomization was necessary as only single variable changed per experiment.
Blinding	X-ray diffraction data were measured quantitatively. In the study blinding was not relevant as we allocated samples into pre-determined experimental groups with the intention of studying the differences between them.

Reporting for specific materials, systems and methods

We require information from authors about some types of materials, experimental systems and methods used in many studies. Here, indicate whether each material, system or method listed is relevant to your study. If you are not sure if a list item applies to your research, read the appropriate section before selecting a response.

Materials & experimental systems

n/a	Involved in the study
[x]	[ ] Antibodies
[x]	[ ] Eukaryotic cell lines
[x]	[ ] Palaeontology and archaeology
[x]	[ ] Animals and other organisms
[x]	[ ] Human research participants
[x]	[ ] Clinical data
[x]	[ ] Dual use research of concern

Methods

n/a	Involved in the study
[x]	[ ] ChIP-seq
[x]	[ ] Flow cytometry
[x]	[ ] MRI-based neuroimaging
