# Supplementary material for: Structural basis for mitoguardin-2 mediated lipid transport at ER-mitochondrial membrane contact sites
Source: Nat Commun. 2022 Jun 28;13:3702. doi: 10.1038/s41467-022-31462-6 (PMC9239997; doi:10.1038/s41467-022-31462-6)
Supplement: Supplementary file 2 — Peer Review File [file 41467_2022_31462_MOESM2_ESM.pdf]

nature portfolio

Peer Review File

Structural basis for mitoguardin-2 mediated lipid transport at ER-mitochondrial membrane contact sites

Open Access This file is licensed under a Creative Commons Attribution 4.0 International License, which permits use, sharing, adaptation, distribution and reproduction in any medium or format, as long as you give appropriate credit to the original author(s) and the source, provide a link to the Creative Commons license, and indicate if changes were made. In the cases where the authors are anonymous, such as is the case for the reports of anonymous peer reviewers, author attribution should be to 'Anonymous Referee' followed by a clear attribution to the source work. The images or other third party material in this file are included in the article's Creative Commons license, unless indicated otherwise in a credit line to the material. If material is not included in the article's Creative Commons license and your intended use is not permitted by statutory regulation or exceeds the permitted use, you will need to obtain permission directly from the copyright holder. To view a copy of this license, visit http://creativecommons.org/licenses/by/4.0/.

Reviewers' Comments:

Reviewer #1:
Remarks to the Author:
The manucscript by Kim H et al. reports the crystal structures of the lipid droplet targeting domain of Xenopus MIGA2 and the MSP domain of mouse VAP-B in complex with a phosphorylated model FFAT motif. The structures convincingly show that MIGA2 is a lipid binding protein and that VAP-B binds the FFAT motif. The biochemical part of the study provides evidence that MIGA2 might function in lipid transfer between organelle membranes. This function is stimulated by membrane tethering through VAP-B.
In general, this is an exciting paper and highly timely. The data is convincing and of good quality. The work is of exceptional interest to the broad readership of Nature Communications and a significant next step in the field. Despite some constructive criticism outlined below the paper is highly recommended for publication pending the clarification of the points that follow.

Major comment:

The increase of lipid transfer activity by the phospho-mimetic MIGA2 mutant is a great result! The interpretation that this is because of increase tethering between the liposomes makes sense, but there is no direct data that shows any kind of tethering between the liposomes. The authors should add dynamic light scattering or TEM data to show that the liposome cluster to different degrees in the S292E/S292D mutant. This would be necessary in order to exclude other unexpected activating effects of the mutant.

Comment on literature citation
The introduction and parts of the results creates the impression as if the formation of MIGA2-VAP contact between the ER and mitochondria was discovered in reference 28. However, the graphical abstract and the data in reference 31 clearly show that the FFAT motif in MIGA2 and its interaction with VAP proteins in the ER was uncovered by the authors of reference 31. Reference 28 showed that the MIGA2 and fly Miga FFAT motif is phosphorylated. The order of citation should be corrected and the text should be changed to reflect the literature correctly (i.e. p4 second paragraph p5 first paragraph has to be corrected, and p10 "It has been shown that MIGA2 can associate with ER membranes via its FFAT motif" change 28 to 31 or mention both together).
Further the work by DiMattia et al. shows that MIGA2 has the strongest prediction score for a phospho-FFAT domain in the human proteome. This paper defines the term phospho-FFAT motif and shows that it is a general feature. Given that the phospho-FFAT motif is so central to the structural work in the study that is under review here, Di Mattia et al. should not only be cited late in the results sections, but should be mentioned in the introduction.

Minor comments:

Fig. 1: Please show the size exclusion chromatography results (in the supplemental information) for the constructs used in the liposome pelleting experiments. It is currently not clear whether the proteins dimerize/oligomerize as the native gels indicate and whether this influences the binding activity that is measured.
Please indicate the molecular weight in kDA for the gels in Fig. 1C.
Please label the lanes in Fig. 1D right panel, and add a molecular weight standard if possible for this method.

Fig. 2: Provide a reference or an explanation for the DALi program and how it was used for the structural "comparison". The areas circled in Fig. 2D are claimed to be lipid binding motifs. It is not easy to see what the authors aim to highlight and a more detailed explanation of how this functional analogy is to be understood is necessary.

Fig. 3A is extremely crowded and it is hard to see the interactions. Many of the labels cannot be associated with structural details; please make this more accessible.
The steric hindrance of lipid binding by the W-mutants is slightly over-interpreted. The data does not show convincingly a "marked" reduction of lipid affinity, and the double mutant does not seem to show a synergistic effect as claimed in the text describing Fig. 3B. Can it be excluded that these mutations lead to folding defects of the hydrophobic cavity? Please comment on this in the text or present gel filtration experiments showing that these mutants are folded correctly and add a molecular weight standard to the gels. Why does the lipid binding essay look so different compared to the mMIGA constructs in Fig. 1?

The data in Fig.4 is beautiful, however the text describing the lipid transfer assay requires revision. It is not clear how the authors can conclude that zMIGA2 is specific for NBD-PE. Is there and evidence for this? Perhaps this is a misunderstanding, which can be easily clarified. A more likely explanation for the increase in NBD fluorescence is that both NBD-PE and Rh-PE are transferred by zMIGA2 and are thus diluted by exchange with the unlabelled acceptor liposomes. The resulting increase in distance between the fluorophores leads to an increase in NBD fluorescence. The drawing should be adjusted accordingly.

Further, to assess the transfer efficiency, it would help to express the data in % of total fluorescence (which is mesaured after the liposomes are treated with large amounts of detergent). It is currently not clear how the measured NBD fluorescence relates to the lipid transport efficiency. Is this a really strong effect, as this reviewer in fact assumes, or is the transport capacity relatively low?
Repeat one of the experiments in Fig. 4B to provide this information.

Regarding the effect of the W-mutants, there is a significant difference between the WT and the W-mutants, but it is not clear if the slight reduction is of functional consequence. Why have the authors not expressed the transport rates relative to WT?
The authors describe the mutants as "lipid binding defective" and say that they are "markedly impaired" in transfer of NBD-PE. These statements should be validated. It is currently not clear whether the measured decrease is indeed indicative of a binding "defect" and "impaired" transport activity or reflect a slight decrease of lipid binding and transfer. This should be addressed.

Fig. 5b could be improved by better labelling. The labelling of the input lanes needs to be clearer. It would also help to label the protein bands (VAP-B and MIGA2 fragment) at the side of the gel.
The interpretation of the Coomassie stained gel is difficult. The SS/ED mutant has clearly the highest affinity to VAP-B MSP, which is a nice result, but the authors say that the WT MIGA2-fragment was not pulled down, although there is clearly a visible band at the right size. Perhaps this is a confusion and it would be necessary to add western blots for this experiment or quantification by densitometry of the bands. The ITC experiments would perhaps be good in the main figure. The really low affinity of WT-MIGA2-fragments that is measured by ITC is more convincing than the pulldowns.

The VAP structure is really interesting and reveals novel insight into the binding of phospho-FFAT motifs. How was the FFAT peptide phosphorylated? Is this relevant for the methods?

Please add molecular weight markers in Fig.6B.

Reviewer #2:
Remarks to the Author:
In this study, Kim et al. present structural and biochemical data indicating that the mitochondrial protein MIGA2, previously identified as a component of a VAPB-dependent ER-mitochondria (and LD) tether, possesses lipid transfer activity selective for phospholipids, specifically PS, and that this

activity is enhanced by its interaction with VAPB tethered to liposomes. The likely lipid transport module of MIGA2 is interesting in that it is similar to APOE and TIP47, but with a large hydrophobic cavity. Data convincingly support the authors' conclusion and thus represent a significant advance to the cell biology field. They also observe that MIGA2 possesses an intrinsic ability to bind liposomes, which is not necessarily expected for a lipid transport protein that extracts lipids from membranes. This is an interesting observation, but it is also the least developed. In terms of functional relevance, cell-based experiments to analyze the impact of the MIGA2 mutants proposed to be selectively deficient in lipid transfer and liposome would improve the manuscript also, but this work may be beyond the scope of the study. The presentation of data within figures could be improved.

Specific comments:

Presentation suggestions: Figure 1 demonstrating liposome binding is out of place as this aspect of MIGA2 is interrogated in Figure 6. Figure 4A, E and F report on in vitro lipid transfer rates and should be combined together so that there is direct comparison with the PE transfer rates presented in A separately.

Scientific comments:
The authors state in their discussion that they "We have established the molecular basis by which MIGA2 is involved in trafficking glycerophospholipids between membranes of the ER, mitochondria, and LDs." The exact nature of the LD connection is not addressed in this study and thus this is an overstatement and should be edited accordingly.

The ability of MIGA2 to bind liposomes is interesting but as stated above the least developed aspect of the study. The authors test a set of mutations in residues of helices that create a positively charged concave surface (R454D/R456D, W457D and K476D/R480D) for both liposome binding and lipid transport. There is no quantification of the liposome binding assay so it is not clear whether the severity of the two phenotypes correlate. In addition, these mutants were not tested for folding defects. At a minimum, to further probe the specificity of the liposome binding defect, the authors should also test the lipid transport mutant (F488W and V430W and the double, which should also be tested for folding) for their ability to bind liposomes. In addition, they should determine what lipids are required for the MIGA2 binding activity. It would also be interesting to test whether MIGA2 is able to bind LDs or a monolayer in vitro recapitulation of LDs.

Does MIGA1 possess liposome binding activity? This would be interesting as the authors report that MIGA1 does not mediate lipid transport. The lack of lipid transport activity of MIGA1 is not commented on further and thus warrants more consideration.

Reviewer #3:
Remarks to the Author:
This excellent manuscript describes thorough structural and functional studies of mitoguardin-2 (MIGA2), an integral membrane protein anchored in the mitochondrial outer membrane that functions at ER-mitochondria contact sites. The work is built around two crystal structures. In the first, the authors determine the structure of the so-called Lipid Droplet-targeting domain (LD) of MIGA2. Although the structure has recognizable resemblance to previously determined structures (APOE and TIP47), it is unique in having a large cup-shaped cavity and a bound glycerophospholipid, presumably PE. This immediately suggests that MIGA2 might be able to function as a phospholipid transfer protein, a hypothesis strongly supported by functional experiments. Also important for lipid transfer is a basic concave surface on the side of the LD, which the authors propose mediates interactions with the endoplasmic reticulum. Intriguingly, functional experiments reveal that the LD has a rather strong preference for phosphatidylserine (PS) over the other lipids tested. Understanding the structural basis for this preference will need to wait, however, because the authors have so far been unable to obtain

MIGA2-PS crystals. In any case, they also determined a second crystal structure, this one containing a MIGA2 peptide bound to the ER protein VAPB. Using phosphomimetic mutants, they demonstrated that double phosphorylation of the MIGA2 peptide, a so-called FFAT motif, is required for tight binding and, in a reconstituted system, for optimal lipid transfer. Overall, this work represents a substantial advance in our understanding of lipid transfer at the ER mitochondria interface, quite suitable for publication in Nature Communications.

I have only minor comments.

1. I would suggest promoting Fig. S7, the authors overall model for MIGA2 function, to a non-supplementary figure. It would seem like a disservice to the reader to hinder access to this nice overview of the authors' findings/model.

2. The consequences of making double Trp mutations in the lipid tail-binding cavity did not seem as severe as I would have expected. And yet there seems little doubt, based on the structure, that this is the site of lipid binding. Perhaps the authors can comment on this.

3. The Kd values quoted in the text and Table S2 should include errors.

4. At the bottom of p. 12, the authors use the word "surprisingly". I found this confusing because the result was just what one would expect if the authors' model were correct. I would leave it out or, possible, substitute with "notably".

5. In Fig. 6b, I was confused by the concentrations across the top – what concentrations are these?

6. The first full paragraph in on p. 16 belongs in the Results section, not the Discussion.

7. Finally, a couple of typos: The authors state on p. 4 that "this interaction requires phosphorylation of two phenylalanines", but of course they mean serines. A second small typo is in the first full paragraph on p. 9, which I believe should read: "…MIGA2 can transfer glycrophopholipids between membranes…" (not across the membrane).

REVIEWER COMMENTS

Reviewer #1 (Remarks to the Author):

The manuscript by Kim H et al. reports the crystal structures of the lipid droplet targeting domain of Xenopus MIGA2 and the MSP domain of mouse VAP-B in complex with a phosphorylated model FFAT motif. The structures convincingly show that MIGA2 is a lipid binding protein and that VAP-B binds the FFAT motif. The biochemical part of the study provides evidence that MIGA2 might function in lipid transfer between organelle membranes. This function is stimulated by membrane tethering through VAP-B.

In general, this is an exciting paper and highly timely. The data is convincing and of good quality. The work is of exceptional interest to the broad readership of Nature Communications and a significant next step in the field. Despite some constructive criticism outlined below the paper is highly recommended for publication pending the clarification of the points that follow.

Major comment:

The increase of lipid transfer activity by the phospho-mimetic MIGA2 mutant is a great result! The interpretation that this is because of increase tethering between the liposomes makes sense, but there is no direct data that shows any kind of tethering between the liposomes. The authors should add dynamic light scattering or TEM data to show that the liposome cluster to different degrees in the S292E/S292D mutant. This would be necessary in order to exclude other unexpected activating effects of the mutant.

⇒ We thank the Reviewer for the positive comments and for pointing this out. As suggested, we performed dynamic light scattering (DLS) experiments to test whether S292E/S292D mutant actually induces the formation of a liposome cluster, and found that unlike wild type His-mMIGA2$^{275–570}$, liposome particle size was increased by approximately 5-fold in the presence of the His-mMIGA2$^{275–570}$ (S292E/S295D) phosphorylation mimic mutant (Fig. 5h). No particle size increases were observed with histag-free mMIGA2$^{275–570}$ or mMIGA2$^{275–570}$ (S292E/S295D) (Fig. 5h), indicating that the liposome cluster was generated by the interaction between phosphorylation mimic mutation of MIGA2 FFAT motif and VAPB, when both were tethered to liposomes.

Comment on literature citation

The introduction and parts of the results creates the impression as if the formation of MIGA2-VAP contact between the ER and mitochondria was discovered in reference 28. However, the graphical abstract and the data in reference 31 clearly show that the FFAT motif in MIGA2 and its interaction with VAP proteins in the ER was uncovered by the authors of reference 31. Reference 28 showed that the MIGA2 and fly Miga FFAT motif is phosphorylated. The order of citation should be corrected and the

text should be changed to reflect the literature correctly (i.e. p4 second paragraph p5 first paragraph has to be corrected, and p10 “It has been shown that MIGA2 can associate with ER membranes via its FFAT motif” change 28 to 31 or mention both together). Further the work by DiMattia et al. shows that MIGA2 has the strongest prediction score for a phospho-FFAT domain in the human proteome. This paper defines the term phospho-FFAT motif and shows that it is a general feature. Given that the phospho-FFAT motif is so central to the structural work in the study that is under review here, Di Mattia et al. should not only be cited late in the results sections, but should be mentioned in the introduction.
⇒ We thank the Reviewer for pointing this out. I agree with the Reviewer’s comment. Based on the Reviewer’s opinion, we have edited the text accordingly, and double checked for any other mistakes throughout the text.

Minor comments:

Fig. 1: Please show the size exclusion chromatography results (in the supplemental information) for the constructs used in the liposome pelleting experiments. It is currently not clear whether the proteins dimerize/oligomerize as the native gels indicate and whether this influences the binding activity that is measured.

⇒ As the Reviewer’s suggested, we have now added the results of size exclusion chromatography and SDS-PAGE (in the Supplementary Fig. 1) for the proteins used in the liposome pelleting experiments. Based on the results, MIGA2 LD domain (residues 313–570) forms a monomer as consistent with our structure. However, other MIGA2 constructs containing the middle domain (residues 161–300) eluted from the SEC column earlier than their calculated molecular weights, indicating that the MIGA2 middle domain forms an oligomer. This observation is consistent with previous data (Zhang *et al*, 2016) showing that MIGA2 forms a homo-dimer or a hetero-dimer with MIGA1.

Please indicate the molecular weight in kDA for the gels in Fig. 1C.

⇒ We have now added the molecular weight markers (kDa) to the gels in Fig. 1c.

Please label the lanes in Fig. 1D right panel, and add a molecular weight standard if possible for this method.
⇒ We have labeled the lanes in the right panel (Fig. 1d). Since this is a native gel, it was difficult to add molecular weight markers.

Fig. 2: Provide a reference or an explanation for the DALi program and how it was used for the structural “comparison”.

⇒ We have now added the reference for the DALI server.

The areas circled in Fig. 2D are claimed to be lipid binding motifs. It is not easy to see what the authors aim to highlight and a more detailed explanation of how this functional analogy is to be understood is necessary.

⇒ We thank the Reviewer for raising this issue. The circles were included in the figure to indicate lipid binding regions. However, the regions shown in APOE and TIP47 are putative lipid binding regions for which there is no direct evidence. Therefore, we have removed all circles from the figure, and focused on comparing the overall structures of MIGA2, APOE, and TIP47.

Fig. 3A is extremely crowded and it is hard to see the interactions. Many of the labels cannot be associated with structural details; please make this more accessible.

⇒ As the Reviewer suggested, we have simplified the labels to make the figure more easily accessible.

The steric hindrance of lipid binding by the W-mutants is slightly over-interpreted. The data does not show convincingly a “marked” reduction of lipid affinity, and the double mutant does not seem to show a synergistic effect as claimed in the text describing Fig. 3B. Can it be excluded that these mutations lead to folding defects of the hydrophobic cavity? Please comment on this in the text or present gel filtration experiments showing that these mutants are folded correctly and add a molecular weight standard to the gels.

⇒ We thank the Reviewer for pointing this out. To exclude the possibility of folding defects caused by mutations, we performed circular dichroism (CD) spectroscopy on the mutant proteins. The CD measurements confirmed that the mutants had no folding defects (Supplementary Fig. 6). In addition, we carried out liposome binding experiments using the mutants and found no effect of the mutations on liposome binding, which means that the reduction of lipid binding of the mutants was due to steric hindrance caused by mutation of the residues involved in lipid binding. As the Reviewer suggested, we have edited the text accordingly. Because of the reason mentioned above, we could not add standard molecular weight markers to the gels.

Why does the lipid binding essay look so different compared to the mMIGA constructs in Fig. 1?

⇒ There was a difference in the percentage of acrylamide in the native gels. We used 10% and 14% acrylamide for native gels in Figs. 1d and 3b, respectively. When we ran the samples using gels with the same percentage of acrylamide, the bands migrated to the same positions (please see below figure). We have added the gel % of acrylamide to the figure legends (Figs. 1d and 3b).

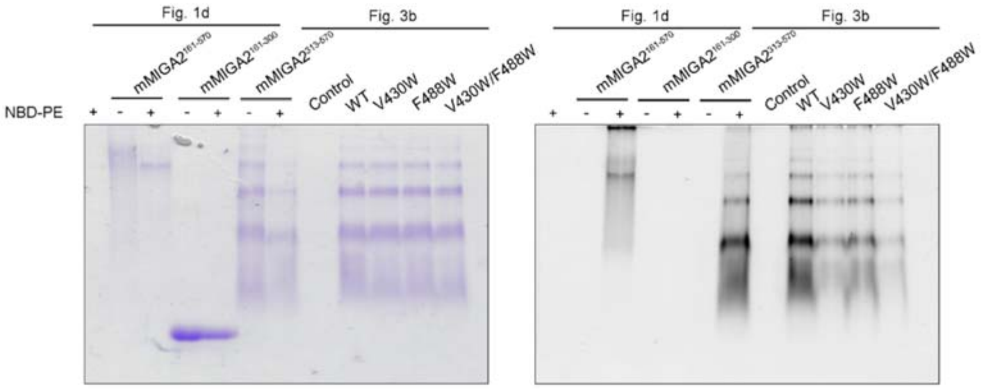

The data in Fig.4 is beautiful, however the text describing the lipid transfer assay requires revision. It is not clear how the authors can conclude that zMIGA2 is specific for NBD-PE. Is there and evidence for this? Perhaps this is a misunderstanding, which can be easily clarified. A more likely explanation for the increase in NBD fluorescence is that both NBD-PE and Rh-PE are transferred by zMIGA2 and are thus diluted by exchange with the unlabelled acceptor liposomes. The resulting increase in distance between the fluorophores leads to an increase in NBD fluorescence. The drawing should be adjusted accordingly.

⇒ We thank the Reviewer for raising this issue. To obtain these data, we performed a general lipid transfer assay according to methods described in previously published papers (Kawano *et al*, 2018; Watanabe *et al*, 2015). For clarity, we have modified the schematic figure (Fig. 4a) and the text to help the reader understand more easily what we mean. Rh-PE could not be transferred by MIGA2 since it has a bulky head group. NBD-cholesterol data (Fig. 4e and Supplementary Fig. 7a) could be a negative control.

Further, to assess the transfer efficiency, it would help to express the data in % of total fluorescence (which is mesaured after the liposomes are treated with large amounts of detergent). It is currently not clear how the measured NBD fluorescence relates to the lipid transport efficiency. Is this a really strong effect, as this reviewer in fact assumes, or is the transport capacity relatively low?
Repeat one of the experiments in Fig. 4B to provide this information.
⇒ As suggested, we have added the data (in % of total fluorescence) to Figure 4b.

Regarding the effect of the W-mutants, there is a significant difference between the WT and the W-mutants, but it is not clear if the slight reduction is of functional consequence. Why have the authors not expressed the transport rates relative to WT? The authors describe the mutants as “lipid binding defective” and say that they are “markedly impaired” in transfer of NBD-PE. These statements should be validated. It is currently not clear whether the measured decrease is indeed indicative of a binding “defect” and “impaired” transport activity or reflect a slight decrease of lipid binding and transfer. This

should be addressed.

⇒ As suggested, we have changed the graph (Fig. 4c) to show the transport rates relative to that of the WT. In the text, we used the phrase “lipid binding defective” to remind the reader of what the mutant (V430W and F488W) is, because we used these mutants to show the lipid binding defect. However, since we also think that this phrase might confuse the reader, we have deleted the phrase from the text.

Fig. 5b could be improved by better labelling. The labelling of the input lanes needs to be clearer. It would also help to label the protein bands (VAP-B and MIGA2 fragment) at the side of the gel.

⇒ We have edited the labels in the figure.

The interpretation of the Coomassie stained gel is difficult. The SS/ED mutant has clearly the highest affinity to VAP-B MSP, which is a nice result, but the authors say that the WT MIGA2-fragment was not pulled down, although there is clearly a visible band at the right size. Perhaps this is a confusion and it would be necessary to add western blots for this experiment or quantification by densitometry of the bands. The ITC experiments would perhaps be good in the main figure. The really low affinity of WT-MIGA2-fragments that is measured by ITC is more convincing than the pulldowns.

⇒ We thank the Reviewer for pointing this out. We think that the visible band (WT MIGA2) pointed out by the Reviewer is probably an impurity since we observed the band in the absence of MIGA2. To make this clear, we have added negative controls (w/o MIGA2) to the gels and indicated the band as an impurity in the figure legend. Furthermore, as the Reviewer suggested, we have included band quantification data of repetitive experiments and have moved the associated ITC data from supplementary data to the main figure (Fig. 5b,c and f).

The VAP structure is really interesting and reveals novel insight into the binding of phospho-FFAT motifs. How was the FFAT peptide phosphorylated? Is this relevant for the methods?

⇒ We used a chemically synthesized phospho-FFAT peptide. The detailed structural analysis of the FFAT-VAPB complex is explained in the methods section.

Please add molecular weight markers in Fig.6B.

⇒ We have added the molecular weight markers to Fig.6b and quantification data to the right panel.

Reviewer #2 (Remarks to the Author):

In this study, Kim et al. present structural and biochemical data indicating that the mitochondrial protein MIGA2, previously identified as a component of a VAPB-dependent ER-mitochondria (and LD) tether, possesses lipid transfer activity selective for phospholipids, specifically PS, and that this activity is enhanced by its interaction with VAPB tethered to liposomes. The likely lipid transport module of MIGA2 is interesting in that it is similar to APOE and TIP47, but with a large hydrophobic cavity. Data convincingly support the authors' conclusion and thus represent a significant advance to the cell biology field. They also observe that MIGA2 possesses an intrinsic ability to bind liposomes, which is not necessarily expected for a lipid transport protein that extracts lipids from membranes. This is an interesting observation, but it is also the least developed. In terms of functional relevance, cell-based experiments to analyze the impact of the MIGA2 mutants proposed to
be selectively deficient in lipid transfer and liposome would improve the manuscript also, but this work may be beyond the scope of the study. The presentation of data within figures could be improved.

Specific comments:

Presentation suggestions: Figure 1 demonstrating liposome binding is out of place as this aspect of MIGA2 is interrogated in Figure 6.

⇒ We thank the Reviewer for pointing this out. I agree with the Reviewer's opinion. However, we wanted to present the liposome binding results of the mutants (lipid binding defective) as negative controls in Figs. 3b and 4c. Therefore, considering the flow of information in the manuscript, we thought it would be better to add the liposome binding results for wild type MIGA2 to Fig. 1.

Figure 4A, E and F report on in vitro lipid transfer rates and should be combined together so that there is direct comparison with the PE transfer rates presented in A separately.

⇒ As the Reviewer suggested, we have added the PE data to Fig. 4e to allow a direct comparison of the PE transfer rates of the various phospholipids.

Scientific comments:
The authors state in their discussion that they "We have established the molecular basis by which MIGA2 is involved in trafficking glycerophospholipids between membranes of the ER, mitochondria, and LDs." The exact nature of the LD connection is not addressed in this study and thus this is an overstatement and should be edited accordingly.

⇒ As suggested, we have deleted "LDs" from the text.

The ability of MIGA2 to bind liposomes is interesting but as stated above the least developed aspect of the study. The authors test a set of mutations in residues of helices that create a positively charged concave surface (R454D/R456D, W457D and K476D/R480D) for both liposome binding and lipid transport. There is no quantification of the liposome binding assay so it is not clear whether the severity of the two phenotypes correlate.

⇒ We have added quantification data to Fig. 6b.

In addition, these mutants were not tested for folding defects.

⇒ To exclude the possibility of folding defects caused by mutations, we performed circular dichroism (CD) spectroscopy on all mutant proteins. The CD measurements confirmed that the mutants had no folding defects (Supplementary Fig. 6a).

At a minimum, to further probe the specificity of the liposome binding defect, the authors should also test the lipid transport mutant (F488W and V430W and the double, which should also be tested for folding) for their ability to bind liposomes.

⇒ We carried out liposome binding experiments using the mutants, and observed no effect of the mutations on liposome binding (Supplementary Fig. 6b), indicating that the reduction in lipid binding by the mutations was due to the steric hindrance caused by mutation of the residues involved in lipid binding. The CD data for F488W and V430W and the V430W/F488W have been added to Supplementary Fig. 6a

In addition, they should determine what lipids are required for the MIGA2 binding activity. It would also be interesting to test whether MIGA2 is able to bind LDs or a monolayer in vitro recapitulation of LDs.

⇒ We thank the Reviewer to raising this issue. As the reviewer suggested, we performed liposome binding experiment using a series of liposomes consisting of various PC-based glycerophospholipid combinations. Interestingly, MIGA2 bound to liposomes containing PA, PS, and PI4P but not PC and PE. These data have been added to Fig. 6c. We tried to test whether MIGA2 binds to LDs or to a lipid monolayer that mimics LDs *in vitro*. However, it was very difficult to obtain stable monolayers or lipid droplets in our system. Therefore, we have removed all remarks concerning lipid droplets from the text.

Does MIGA1 possess liposome binding activity? This would be interesting as the authors report that MIGA1 does not mediate lipid transport. The lack of lipid transport activity of MIGA1 is not commented on further and thus warrants more consideration.

⇒ We thank the Reviewer for pointing this out. First, we carried out liposome binding assays using mouse MIGA1, which revealed that MIGA1 also interacts with liposomes (Supplementary Fig. 8a). Next, we tested whether MIGA1 interacts with individual phospholipids using NBD-PE. Interestingly, MIGA1 showed markedly lower lipid binding than MIGA2 (Supplementary Fig. 8b). Lastly, we performed a lipid extraction experiment as shown in Fig. 1e and f, and found that MIGA1 cannot extract phospholipids from liposomes (Supplementary Fig. 8c). Based on these observations, we concluded that MIGA1 cannot transfer phospholipids because MIGA1 has very low affinity for phospholipids and no ability to extract lipids.

Reviewer #3 (Remarks to the Author):

This excellent manuscript describes thorough structural and functional studies of mitoguardin-2 (MIGA2), an integral membrane protein anchored in the mitochondrial outer membrane that functions at ER-mitochondria contact sites. The work is built around two crystal structures. In the first, the authors determine the structure of the so-called Lipid Droplet-targeting domain (LD) of MIGA2. Although the structure has recognizable resemblance to previously determined structures (APOE and TIP47), it is unique in having a large cup-shaped cavity and a bound glycerophospholipid, presumably PE. This immediately suggests that MIGA2 might be able to function as a phospholipid transfer protein, a hypothesis strongly supported by functional experiments. Also important for lipid transfer is a basic concave surface on the side of the LD, which the authors propose mediates interactions with the endoplasmic reticulum. Intriguingly, functional experiments reveal that the LD has a rather strong preference for phosphatidylserine (PS) over the other lipids tested. Understanding the structural basis for this preference will need to wait, however, because the authors have so far been unable to obtain MIGA2-PS crystals. In any case, they also determined a second crystal structure, this one containing a MIGA2 peptide bound to the ER protein VAPB. Using phosphomimetic mutants, they demonstrated that double phosphorylation of the MIGA2 peptide, a so-called FFAT motif, is required for tight binding and, in a reconstituted system, for optimal lipid transfer. Overall, this work represents a substantial advance in our understanding of lipid transfer at the ER mitochondria interface, quite suitable for publication in Nature Communications.

I have only minor comments.

1. I would suggest promoting Fig. S7, the authors overall model for MIGA2 function, to a non-supplementary figure. It would seem like a disservice to the reader to hinder access to this nice overview of the authors' findings/model.

⇒ As suggested, we have moved Fig. S7 to the main figure (Fig. 7).

2. The consequences of making double Trp mutations in the lipid tail-binding cavity did not seem as severe as I would have expected. And yet there seems little doubt, based on the structure, that this is the site of lipid binding. Perhaps the authors can comment on this.

⇒ The interaction between the hydrocarbon chains of phospholipids and their interacting residues in MIGA2 mainly depends on hydrophobic interactions. The hydrocarbon chains of phospholipids are sufficiently structurally flexible to accommodate the interactions. Although we tried to inhibit the interaction by using a bulky (Trp) side chain to introduce steric hindrance, the effect of the mutation might be less severe than expected, because the Trp side chain is also hydrophobic and hydrocarbon chain of lipids would be structurally flexible. As the reviewer suggested, we have edited the text accordingly.

3. The Kd values quoted in the text and Table S2 should include errors.

⇒ We have added the errors to the text and Table S2.

4. At the bottom of p. 12, the authors use the word "surprisingly". I found this confusing because the result was just what one would expect if the authors' model were correct. I would leave it out or, possible, substitute with "notably".

⇒ We have replaced the word "surprisingly" with “notably” in the text.

5. In Fig. 6b, I was confused by the concentrations across the top – what concentrations are these?

⇒ These are the concentrations of liposomes used in the experiments. We have replaced this label with a symbol indicating increasing concentrations. We have also added quantification data to the right panel.

6. The first full paragraph in on p. 16 belongs in the Results section, not the Discussion.

⇒ This paragraph mainly describes our putative model. Since the experiments provided only indirect evidence, we would prefer to leave this paragraph here to stimulate future work into proving the validity of the model.

7. Finally, a couple of typos: The authors state on p. 4 that "this interaction requires phosphorylation of two phenylalanines", but of course they mean serines. A second small typo is in the first full paragraph on p. 9, which I believe should read: "…MIGA2 can transfer glycrophopholipids between membranes…" (not across the membrane).

⇒ We thank the Reviewer for raising this issue. “two phenylalanines in an acidic tract” is the full name of the FFAT motif. So as not to confuse the reader, we have changed “two phenylalanines in an acidic tract (FFAT) motif” to “FFAT (two phenylalanines in an acidic tract) motif”. We have also changed “across the membrane” to “between membranes”.

Reference

Kawano S, Tamura Y, Kojima R, Bala S, Asai E, Michel AH, Kornmann B, Riezman I, Riezman H, Sakae Y *et al* (2018) Structure-function insights into direct lipid transfer between membranes by Mmm1-Mdm12 of ERMES. *J Cell Biol* 217: 959-974

Watanabe Y, Tamura Y, Kawano S, Endo T (2015) Structural and mechanistic insights into phospholipid transfer by Ups1-Mdm35 in mitochondria. *Nat Commun* 6: 7922

Zhang Y, Liu X, Bai J, Tian X, Zhao X, Liu W, Duan X, Shang W, Fan HY, Tong C (2016) Mitoguardin Regulates Mitochondrial Fusion through MitoPLD and Is Required for Neuronal Homeostasis. *Mol Cell* 61: 111-124

Reviewers' Comments:

Reviewer #1:
Remarks to the Author:
The authors have addressed all comments by this reviewer and there are no further requests. This is an excellent paper and it is ready for publication.

Reviewer #2:
None

Reviewer #3:
Remarks to the Author:
The authors have addressed all of my concerns.
